# The LEDGIN GS-9822 inhibits HIV-2 infection and enhances HIV-2 latency

**DOI:** 10.1128/spectrum.01699-25

**Published:** 2026-04-27

**Authors:** Anne Bruggemans, Gerlinde Vansant, Eline Pellaers, Paulien Van de Velde, Frauke Christ, Zeger Debyser

**Affiliations:** 1 ADVANTAGE: Advanced Disease Modelling, Targeted Drug Discovery and Gene Therapy, Department of Pharmaceutical and Pharmacological Sciences, KU Leuven 522753 , Leuven, Belgium; National Institute of Immunology, New Delhi, India

**Keywords:** HIV-1, HIV-2, LEDGIN, LEDGF, small molecule, inhibitor, SIV, latency, virus

## Abstract

HIV integration is targeted toward active transcription units through the interaction of HIV integrase with the cellular co-factor LEDGF/p75. LEDGINs, small molecule inhibitors of this interaction, reduce HIV-1 integration and retarget residual proviruses away from active transcription units, resulting in a more latent phenotype. LEDGF/p75 interacts with all lentiviral integrases, but early-generation LEDGINs are not effective against HIV-2 or SIV, as they carry a methionine instead of an alanine at the LEDGIN interaction spot. The LEDGIN GS-9822 (Gilead Sciences) was optimized for activity against the HIV-1 integrase A128T LEDGIN resistance mutation, which we hypothesized could confer activity against HIV-2 and SIV. Using AlphaScreen, we show that GS-9822 blocks the interaction of HIV-2 and SIV integrase with LEDGF/p75. In cell culture, we show preliminary data that GS-9822 achieves nanomolar activity against HIV-2 and SIV replication. Using HIV-2 OGH, a single round double reporter construct that can distinguish between latently and productively transduced cells, we show that GS-9822 inhibits integration and that residual HIV-2 integrants are more latent and refractory to reactivation. Finally, using next-generation sequencing, we showed that GS-9822 retargets HIV-2 integration away from active transcription units and toward a more repressive epigenetic chromatin environment. We demonstrate for the first time that LEDGINs can inhibit HIV-2 integration and replication. As GS-9822 shows a difficult-to-monitor urothelial toxicity in non-human primates, future research with new LEDGIN congeners should look into the effects of LEDGINs on HIV-2 and SIV, as this could pave the way for non-human primate studies on the “block and lock” effect of LEDGINs.

**IMPORTANCE:**

HIV latent reservoirs are the major hurdle preventing HIV cure. LEDGINs could be used as part of a “block-and-lock” functional cure strategy to induce a state of deep latency in HIV-1 reservoirs and prevent viral rebound in the absence of antiretroviral therapy. Previous generations of LEDGINs were not active against SIV, and therefore, they could not be used to test this strategy in non-human primate (NHP) models. Our study is the first to show the block-and-lock effects of LEDGINs against HIV-2 *in vitro* and shows the preliminary data of activity against SIV as well. Although GS-9822 itself cannot be used in NHP models, due to urothelial toxicity, this means it is possible to design new LEDGINs with SIV activity for future experiments with the “block-and-lock” strategy in non-human primate models. In addition, we show that LEDGINs can achieve their latency-promoting effects against HIV-2 as well.

## INTRODUCTION

To date, combination antiretroviral therapy (cART) can suppress HIV-1 replication below detectable levels but cannot eliminate the virus from the body because HIV integrates a copy of viral DNA into the genome of the host cells it infects. Latent proviruses in long-lived memory cells that are maintained through homeostatic proliferation or increased through antigenic proliferation or clonal expansion avoid viral cytopathic effects and immune-mediated killing, allowing HIV to persist for decades (1–3). Therefore, the understanding of HIV reservoir formation and the development of treatment strategies targeting proviruses have been defined as key research priorities for an HIV cure (4).

The proviral integration site and the surrounding chromatin environment affect the transcriptional state and proviral latency (5–9). HIV has evolved to preferentially target integration toward active transcription units in gene-dense regions, with markers of active chromatin (6–8, 10–12) through the interaction of HIV integrase with the cellular co-factor Lens Epithelium Derived Growth Factor (LEDGF/p75), among other mechanisms. LEDGF/p75 is a transcriptional co-activator that reads H3K36me2/3 histone modifications with its N-terminal PWWP domain (13, 14), interacts with DNA through its AT-hook-like domain (15, 16), and binds to other cellular proteins or lentiviral integrases with a C-terminal domain called the integrase binding domain or IBD (16–18). During HIV infection, LEDGF/p75 IBD thus tethers the HIV preintegration complex (PIC) to the chromatin, targets HIV integration toward active transcription units (19–22), and stimulates the integration reaction (16). LEDGINs are small molecules that bind to the **LEDG**F/p75 binding site of HIV **IN**tegrase and block the integrase-LEDGF/p75 interaction, which in turn inhibits HIV-1 integration (early effects of LEDGINs) (23–26). In addition, it is important to note that LEDGINs also affect later stages of HIV replication (late effects of LEDGINs). Indeed, the interaction of LEDGINs with the integrase catalytic core domain promotes premature integrase multimerization during viral assembly (27). This causes capsid malformation and displacement of the viral genome outside of the viral capsid core, which potently reduces viral infectivity. Notably, the late effects of LEDGINs tend to occur at lower concentrations than are necessary for the early effects, although the difference in concentration can differ per compound (27–30).

Finally, as the LEDGF/p75 binding site is located away from the catalytic site, which is targeted by integrase strand transfer inhibitors (INSTIs), they represent a separate class of allosteric integrase inhibitors (31). No cross-resistance between INSTIs and LEDGINs was observed, and their combination even showed synergistic effects (32, 33). Although other names for these compounds have been proposed based on these additional characteristics, we still prefer the term “LEDGINs” (23), as the first compounds belonging to this class were published as LEDGINs (24), and all compounds to date share the LEDGF/p75 binding site of HIV IN.

Through the interruption of the IN-LEDGF/p75 interaction (early effect), LEDGINs have been shown to reduce HIV-1 integration (19–21, 34, 35) and retarget residual proviruses away from active transcription units (8, 36–38), resulting in a more repressive epigenetic landscape surrounding the HIV integration site (8, 36, 38). In addition, using a replication-deficient dual reporter virus, HIV-1 OGH (9, 39, 40), residual integrants after LEDGIN treatment were shown to be more latent and more resistant to reactivation (36, 38). These latency-promoting effects of LEDGINs make them powerful tools for studying HIV latency and reactivation. Additionally, since the latent reservoir may be established around the start of cART (41), LEDGINs could be used as part of a “block-and-lock” functional cure strategy. Here, the goal is not to fully eradicate HIV from the body, but to achieve a state of deep latency in residual HIV reservoirs and to obtain an HIV remission with viral suppression even in the absence of cART. However, while LEDGINs have moved to clinical testing for their safety and antiviral effects (42, 43), no *in vivo* data on the latency-inducing effects of LEDGINs are available to date.

LEDGINs were designed to interrupt the LEDGF/p75-HIV-1 IN interaction specifically, and previous generations of LEDGINs were not effective against HIV-2 or SIV (23, 27, 44), which has hampered studies in non-human primate models. An important interaction site for both LEDGF/p75 and LEDGINs is the A128 residue, and a mutation to threonine at this position (A128T) results in LEDGIN resistance *in vitro* (23, 45). SIV and HIV-2 naturally carry a methionine instead of an alanine at this position, preventing binding of the 2-(quinolin- 3-yl)acetic acid derivatives (23). On the other hand, LEDGF/p75 does interact with all lentiviral integrases, including those of SIV and HIV-2 (46, 47), and small peptides binding the IBD of LEDGF/p75 were able to delay HIV-2 infection (48). In addition, similar to HIV-1, SIV, and HIV-2 prefer integration sites in actively transcribed genes and gene-dense regions (10, 48–54).

As part of their goal to develop a novel antiretroviral agent with the potential for low-dose, unboosted, once-daily oral dosing and a high barrier to resistance, Gilead screened representatives from the LEDGIN library against a virus harboring the HIV-1 IN mutations A124T and A128T and the LEDGIN resistance mutation T174I (55), which resulted in the identification of GS-9822 (55). As GS-9822 was optimized for activity against the A128T resistance mutation, we hypothesized that GS-9822 could have increased activity against HIV-2 and SIV replication as well. Although the development of GS-9822 has been halted due to the occurrence of a difficult-to-monitor urothelial toxicity in non-human primates, it was interesting to test this hypothesis *in vitro*.

In this study, we investigated the effect of GS-9822 on the interaction of HIV-2 and SIV IN with LEDGF/p75. We compared the antiviral potency of GS-9822 against HIV-2 and SIV with CX14442 (33), an early-generation research compound. We recently developed HIV-2 OGH, a single-round double reporter construct that allows discrimination between latently and productively transduced cells (9, 39, 40). Using this construct, we investigated the effects of GS-9822 on HIV-2 latency and reactivation. Finally, we used next-generation sequencing to study whether GS-9822 retargets HIV-2 integration. We provide the first evidence that the LEDGIN GS-9822 achieves nanomolar activity against HIV-2 and SIV replication *in vitro*. Much like HIV-1, GS-9822 inhibited HIV-2 integration, and residual integrants were more latent and refractory to reactivation. Finally, GS-9822 retargeted HIV-2 integration away from genes and gene-dense regions and toward a more repressive epigenetic environment. This study proves for the first time that LEDGINs inhibit HIV-2 and SIV integration and replication.

## RESULTS

### GS-9822 potently inhibits the interaction between HIV-1, HIV-2, and SIV integrase and LEDGF/p75

**Fig 1:**
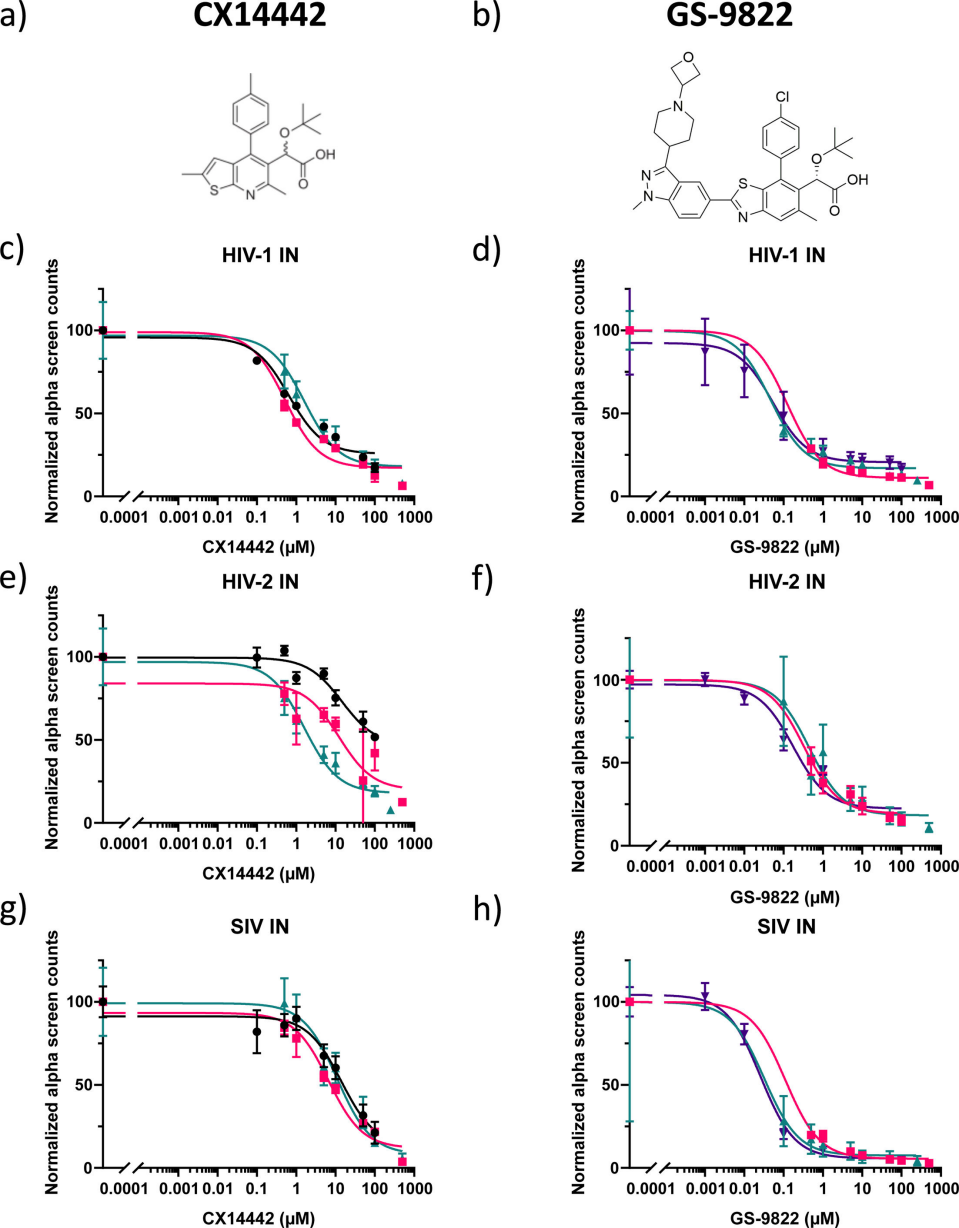
GS-9822 potently inhibits the interaction between the integrases of HIV-1, HIV-2, or SIV and LEDGF/p75. (**a and b**) Chemical structures of CX14442 and GS-9822. (**c–h**) Dose-response curves of CX14442 and GS-9822 in AlphaScreen. Increasing concentrations of compound were added to a mixture of 100 nM LEDGF/p75 and 50 nM of HIV-1 integrase (IN) (strain NL4.3) (**c and d**), 50 nM of HIV-2 IN (strain ROD) (**e and f**), or 50 nM of SIV IN (strain MAC) (**g and h**). Each data point represents the mean and standard deviation from one experiment, and data from three experiments are shown (one color and symbol per experiment), with each experiment performed in duplicate. Data are plotted as a percentage of the signal in the no-drug control. IC50 values are listed in Table 1.

To verify whether GS-9822 inhibits HIV-2 and SIV replication, we first evaluated whether GS-9822 inhibits the interaction between HIV-2 or SIV integrase and LEDGF/p75, as was previously demonstrated for HIV-1 integrase (38). We performed AlphaScreen interaction assays with His6-tagged HIV-1, HIV-2, and SIV integrase and Flag-tagged LEDGF/p75 (23) in the presence of increasing concentrations of the LEDGINs CX14442 or GS-9822 (Fig. 1a and b). Data for HIV-1 IN were obtained in parallel for direct comparison (38). For both compounds, a dose-dependent inhibition was evidenced for all three integrases, with GS-9822 being more potent in each case (Fig. 1c through h; Table 1). While submicromolar concentrations of CX14442 were needed to inhibit the interaction of LEDGF/p75 with HIV-1 IN (IC_50_ = 0.92 ± 0.34 µM) and micromolar concentrations were needed for HIV-2-IN and SIV-IN (IC_50_ = 9.42 ± 3.98 µM and IC_50_ = 10.57 ± 2.16 µM, respectively), nanomolar concentrations of GS-9822 inhibited the interactions of all three integrases with LEDGF/p75 (IC_50_ = 0.07 ± 0.02 µM, IC_50_ = 0.32 ± 0.08 µM, and IC_50_ = 0.06 ± 0.03 µM, for HIV-1, HIV-2, and SIV IN, respectively). GS-9822 was 13-fold, 29-fold, and 176-fold more effective at inhibiting the IN-LEDGF/p75 interaction than CX14442 for HIV-1, HIV-2, and SIV IN, respectively.

**TABLE 1:** hrPotency of CX14442 and GS9822 in antiviral and protein-protein interaction assays^*f*^

	AlphaScreen	MTT/MT-4 assay						
HIV-1	LEDGF/p75 – IN IC_50_^*a*,*g*^	CC_50_^*b*,*h*^	IIIb			NL4.3		
			EC_50_^*c*,*g*^	EC_90d_^*d*,*g*^	SI ^*e*^^,*h*^	EC_50_^*c*,*h*^	EC_90d_^*d*,*h*^	SI ^*e*^^,*h*^
CX14442	0.92 ± 0.34	70.832 ± 7.022	0.051 ± 0.004	0.814 ± 0.594	1.389	0.296 ± 0.135	0.623 ± 0.345	239
GS-9822	0.07 ± 0.02	4.7570 ± 0.5972	0.0022 ± 0.0003	0.0928 ± 0.0593	2.162	0.0025 ± 0.0008	0.0042 ± 0.0011	1903
	**AlphaScreen**	**MTT/MT-4 assay**						
HIV-2	LEDGF/p75 – IN IC_50_^*a*,*g*^	CC_50_^*b*,*h*^	EHO			ROD		
			EC_50_^*c*^	EC_90_^*d*^	SI ^*e*^^,*h*^	EC_50_^*c*,*g*^	EC_90d_^*d*,*g*^	SI ^*e*^^,*h*^
CX14442	9.42 ± 3.98	70.832 ± 7.022	> 17.9^*g*^	> 17.9^*g*^	Nd^*f*^	11.250 ± 0.432	22.240 ± 0.380	6
GS-9822	0.32 ± 0.08	4.7570 ± 0.5972	0.305 ± 0.086^*h*^	> 1.250**h	16	0.077 ± 0.021	0.325 ± 0.070	62
	**AlphaScreen**	**MTT/MT-4 assay**						
SIV	LEDGF/p75 – IN IC_50_^*a*,*g*^	CC_50_^*b*,*h*^	MAC251					
			EC_50_^*c*^	EC_90_^*d*^	SI ^*e*^^,*h*^			
CX14442	10.57 ± 2.16	70.832 ± 7.022	> 17.9^*g*^	> 17.9^*g*^	Nd^*f*^			
GS-9822	0.06 ± 0.03	4.7570 ± 0.5972	0.293 ± 0.117^*h*^	0.508 ± 0.256^*h*^	16			

^*a*^ Inhibitory concentration (µM) required to inhibit the in vitro protein-protein interaction by 50%.

^*b*^ Cytotoxic concentration (µM) reducing cell viability by 50%.

^*c*^ Effective concentration (µM) required to reduce HIV-1 induced cytopathic effects by 50%.

^*d*^ Effective concentration (µM) required to reduce HIV-1 induced cytopathic effects by 90%.

^*e*^ Selectivity index: CC_50_/EC_50._

^*f*^ Not determined.

^*g*^ Mean and SEM of at least three independent experiments.

^*h*^ Mean and SEM of two independent experiments.

### GS-9822 has potent antiviral activity against HIV-1, HIV-2, and SIV replication in cell culture

Next, the EC_50_ of CX14442 and GS-9822 against replication of HIV-1, HIV-2, and SIV was determined by infecting MT4 cells with HIV-1 subtype B viruses (IIIb and NL4.3), HIV-2 subtype A (ROD), and subtype B (EHO) viruses and SIVmac (MAC251) (Table 1). Once again, the data for HIV-1 were obtained in parallel (38). As previously described, the CC_50_ value of GS-9822 is about 15-fold lower than that of CX14442 in these cells (38). For HIV-1, GS-9822 was 23-fold more potent against the IIIb strain and over 100-fold more potent against NL4.3 than CX14442, with an EC_50_ in the low nanomolar range for each strain. For HIV-2 and SIV, GS-9822 achieved an EC_50_ below 0.3 µM for all strains and viruses tested, while concentrations over 10 µM were needed for CX14442. For HIV-2 ROD, GS-9822 was 146-fold more potent than CX14442 and had a 10-fold higher selectivity index (SI, CC_50_/EC_50_). As an additional measure of inhibition of viral replication, we evaluated the effect of GS-9822 on HIV-1, HIV-2, and SIV replication by monitoring p24 in the supernatant during multiple round replication (Fig. S1). We confirm that GS-9822 potently inhibits the replication of HIV-2 and SIVmac in cell culture. At a concentration of 0.05 µM, GS-9822 inhibited HIV-1 replication (Fig. S1a), but not HIV-2 (Fig. S1b) or SIV (Fig. S1c) replication. However, at a concentration of 1 µM, GS-9822 significantly reduced HIV-1 (Fig. S1a), HIV-2 (Fig. S1b), and SIV (Fig. S1c) replication.

### GS-9822 inhibits HIV-2 integration, reduces reporter gene expression, and increases immediate latency of HIV-2-based single-round reporter vectors

**Fig 2:**
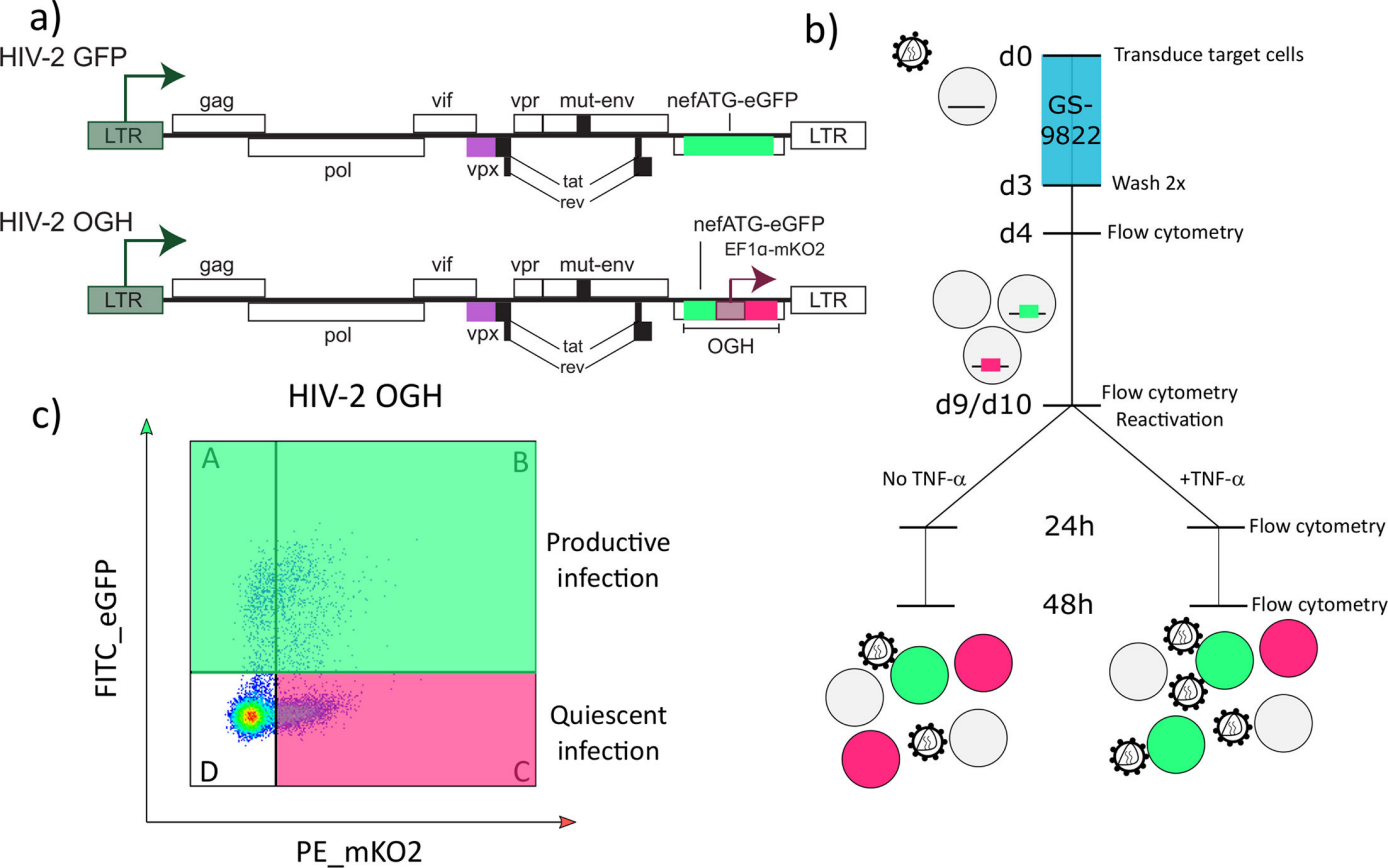
A double reporter construct to analyze the impact of LEDGINs on immediate latency and reactivation. (**a**) Schematic representation of the HIV-2 OGH construct, based on the principles of a previous HIV-1 OGH construct (9, 39, 40, 57). HIV-2 OGH is a replication-deficient virus containing an eGFP gene under control of the viral LTR promoter. HIV-2 OGH also carries a constitutively active transcriptional unit of a mKO2 reporter driven by an EF1α promoter. (**b**) Timeline of the transduction and reactivation experiments. SupT1 cells were transduced in the presence of GS-9822. Three days post-transduction, the virus and compounds were washed away, and flow cytometry analysis was performed on days 3 (data not shown) and 4. On day 9 or 10 post-transduction, the cells were reactivated with 10 ng/mL TNFα. At 24 and 48 h post-reactivation, the cells were harvested for flow cytometry, and the samples were taken for p24 ELISA. (**c**) Representative dot plot 7 days after transduction of 1 × 10^5^ SupT1 cells with 1.10 × 10^4^ pg of HIV-2 OGH, showing how flow cytometry allows us to distinguish between different cell populations. Cells only expressing mKO2-positive cells from the constitutively active EF1α promoter have an inactive LTR and are thus considered to be latently transduced (quadrant C). If cells are productively transduced (quadrant B), the viral LTR promoter will drive eGFP expression.

Since GS-9822 inhibits the interaction between LEDGF/p75 and HIV-2 integrase, we verified the inhibition of HIV-2 integration. We recently demonstrated that LEDGIN treatment with CX14442 and GS-9822 can reduce transcription, increase immediate latency, and reduce the reactivation capacity of HIV-1 (8, 36, 38). To study if GS-9822 induces similar effects to HIV-2, we used the reporter viruses HIV-2 eGFP and HIV-2 OGH (Fig. 2a) (56). Both constructs are near full-length, HIV-2 ROD-based viruses that are replication-deficient due to a deletion in the *env* region. Except for *env* and *nef*, all other structural and accessory HIV-2 genes remain intact. HIV-2 eGFP contains an eGFP gene in the *nef* position, under control of the viral LTR. HIV-2 OGH was cloned based on a similar construct for HIV-1 (HIV-1 OGH) and was described previously (9, 39, 40, 57). HIV-2 OGH contains a dual reporter cassette inserted in the *nef* position. It contains an eGFP reporter gene, driven by the LTR promoter, whereas a constitutively active EF1α promoter drives mKO2, a second reporter. This dual-reporter system allows the use of flow cytometry to distinguish different cell populations. If a cell is productively infected, LTR activity will result in eGFP expression, whereas quiescently infected cells will only express mKO2 (Fig. 2c). As previously described for both HIV-1 OGH and HIV-2 OGH, a small percentage of transduced cells express eGFP but not mKO2 (9, 39, 40, 56). Since these cells do express eGFP, they are considered to be actively transduced (39, 40, 56). For each experiment, we plotted the percentage of transduced positive cells (eGFP or mKO2 positive, Fig. 2c quadrant A+B + C) and the percentage of eGFP-positive cells (Fig. 2c quadrant A+B). In addition, the OGH construct allows us to calculate the latent fraction (single mKO2-positive cells/transduced-positive cells or quadrant C/quadrant A+B+C in Fig. 2c).

**Fig 3:**
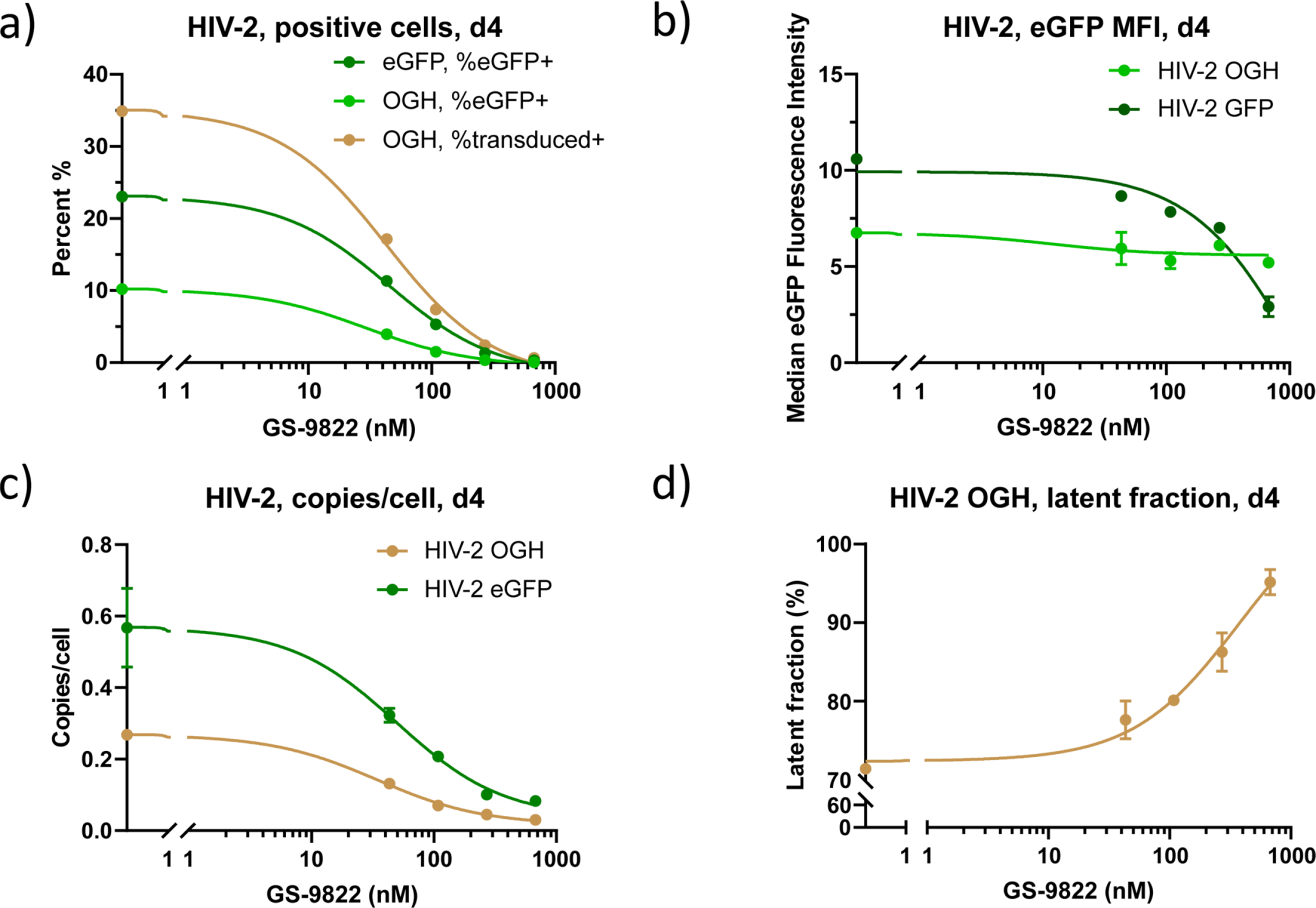
Treatment with GS-9822 inhibits transduction, decreases expression, and increases immediate latency of the HIV-2 OGH double reporter construct. (**a–d**) In total, 7.5 × 10^5^ SupT1 cells were transduced with 2.48 × 10^4^ pg of HIV-2 OGH or 1.00 × 10^5^ pg of HIV-2 eGFP in the presence of increasing concentrations of GS-9822. Data were measured on day 4; one representative experiment out of 7 is shown. The averages of duplicate measurements with standard deviations are shown. (**a**) Percentage of eGFP-positive (eGFP+) and transduced-positive cells (eGFP- and/or mKO2-positive; transduced+), (**b**) median eGFP fluorescence intensity (eGFP MFI), and (**c**) copies per cell as calculated by HIV-2 Gag qPCR and CCR5 qPCR, (**d**) Latent fraction [percentage of single mKO2 positive cells/(percentage of transduced+)*100] or [quadrant C/(quadrant A+B+C)] as shown in Fig. 2a–2c of SupT1 cells transduced with HIV-2 OGH.

We transduced 1x10^5^ SupT1 cells with 3.30–4.40 × 10^3^ pg of HIV-2 OGH or 1.33–1.78 × 10^4^ pg of HIV-2 eGFP in the presence of increasing concentrations of GS-9822 for 3 days, after which viruses and compound were washed away (Fig. 2b). We performed a first flow cytometry analysis 4 days post-transduction. GS-9822 indeed inhibited HIV-2 transduction in a concentration-dependent manner, as measured by the percentage of transduced-positive and eGFP-positive cells for each HIV-2 virus (Fig. 3a; Fig. S2a and e). We measured an IC_50_ of 33.49 ± 3.756 nM for HIV-2 OGH and 39.61 ± 4.584 nM for HIV-2 eGFP. The decrease in the percentage of positive cells correlated with a decrease in HIV-2 copy numbers per cell (Fig. 3c; Fig. S2d and g), with an IC_50_ of 36.56 ± 12.20 nM for HIV-2 OGH and 23.74 ± 5.854 nM for HIV-2 eGFP. In addition, at concentrations above 100 nM, a decrease in the eGFP median fluorescent intensity (eGFP MFI) was evidenced for both constructs (Fig. 3b; Fig. S2b and f), pointing to reduced expression of this reporter protein. Finally, for HIV-2 OGH, an increase in the latent fraction was evidenced at concentrations above 100 nM (Fig. 3d; Fig. S2c).

These results show that GS-9822 inhibits HIV-2 integration, reduces the expression of eGFP driven by the HIV-2 LTR, and increases immediate latency, similar to what was previously reported for HIV-1 (8, 36, 38).

### GS-9822, when added during infection, makes residual HIV-2 proviruses more resistant to reactivation

**Fig 4:**
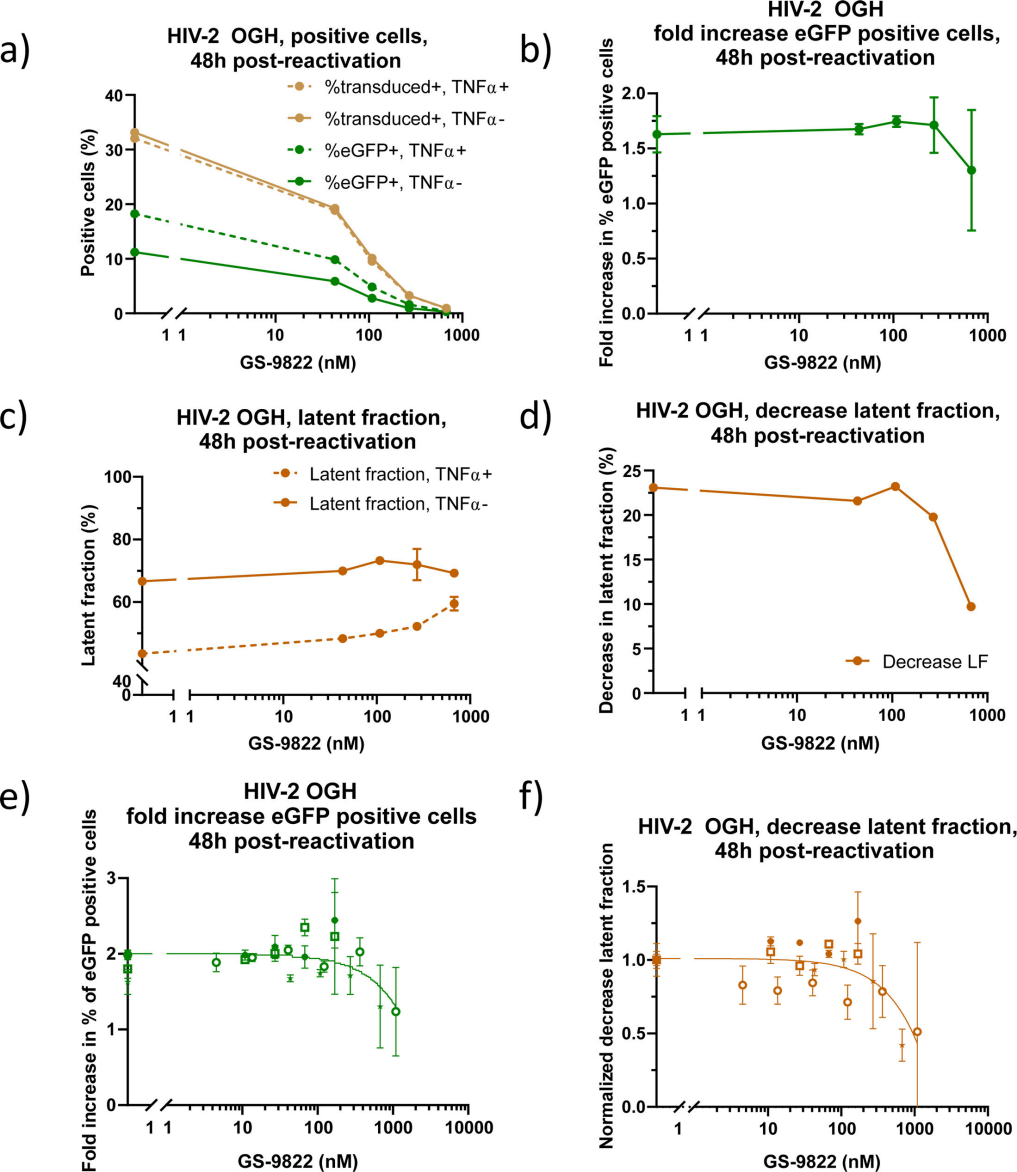
GS-9822, when added during infection, results in residual HIV-2 proviruses more resistant to reactivation. (**a–f**) Flow cytometry data on day 12 or 48 h after reactivation with 10 ng/mL TNFα of SupT1 cells previously transduced with HIV-2 OGH (750,000 cells and 2.48 × 10^4^ pg). Cells were pre-treated with increasing concentrations of GS-9822. Full lines represent non-activated cells, and dotted lines represent TNFα-treated cells. Data show one representative experiment out of three, and the averages of duplicate measurements with standard deviation are plotted. (**a**) Percentage of transduced-positive (eGFP- or mKO2-positive cells) and eGFP+ cells. (**b**) Fold increase in eGFP+ cells (%eGFP+ TNFα-treated cells/ %eGFP+ untreated cells). (**c**) The latent fraction, [percentage of single mKO2+ cells/(percentage of transduced+ cells)*100] or [quadrant C/(quadrant A+B+C)] as shown in Fig. 2c in TNFα-treated and -untreated cells. (**d**) Upon reactivation, the latent fraction decreases, and the plotted decrease in latent fraction is calculated by subtracting the latent fraction in the TNFα-treated condition from the latent fraction in the non-treated condition. (**e–f**) Summary data of 4 experiments of 1 × 10^5^ SupT1 cells transduced with 2.76–4.41 × 10^3^ pg of HIV-2 OGH and pre-treated with GS-9822. Each data point represents the mean and SD from one experiment, with one color per experiment. Transduction was done in the same way for all experiments, but for one experiment, reactivation was performed on day 8 instead of day 10. (**e**) Fold increase in eGFP-positive cells, calculated as previously described. (**f**) Decrease in latent fraction was calculated as previously described. All values were normalized to the decrease in latent fraction in the no-drug control.

Next, we evaluated HIV-2 reactivation in SupT1 cells pre-treated with GS-9822. We transduced 1 × 10^5^ SupT1 cells with 2.76–4.40 × 10^3^ pg of HIV-2 OGH or 5.55 × 10^3^ to 1.52 × 10^4^ pg HIV-2 eGFP vector for 3 days in the presence of increasing concentrations of GS-9822, then washed twice to remove compound and cell-free virus and kept cells in culture until day 10 in the absence of inhibitor. On day 10 post-transduction, half of the cells were reactivated with 10 ng/mL TNFα, and the other half were left untreated. No additional GS-9822 was added at this point. Cells were harvested for flow cytometry on days 11 and 12 or 24 and 48 h post-reactivation, respectively (Fig. 2b). Upon reactivation, the percentage of transduced-positive cells remained stable for HIV-2 OGH as expected, while the percentage of eGFP-positive cells increased for both HIV-2 OGH and HIV-2 eGFP (Fig. 4a; Fig. S3a and S4a). However, reactivation, as measured by the fold increase in eGFP-positive cells, seemed to show a decreasing trend in cells pre-treated with increasing concentrations of GS-9822 for HIV-2 OGH as measured at 48 h (Fig. 4b and e: data from one experiment and summary data from four experiments, respectively) and to a lesser degree at 24 h (Fig. S3b and e), although this was not evidenced for HIV-2 eGFP (Fig. S4b through d). Upon reactivation of cells transduced with HIV-2 OGH, the latent fraction decreased, due to cells being reactivated from latency and resuming eGFP expression (Fig. 4c; Fig. S3c). For cells pre-treated with GS-9822, a dose-dependent decrease in this reactivatable latent fraction was evidenced at 48 h (Fig. 4d and f: data from one experiment and summary data from four experiments, respectively) and to a lesser degree at 24 h (Fig. S3d and f). Previously, we demonstrated that HIV-2-transduced cells require 48 h to reactivate HIV expression, which may explain the results obtained (56).

Overall, the flow cytometry data seem to point to the generation of an HIV-2 reservoir that is more refractory to reactivation after treatment with GS-9822, similar to the effect of GS-9822 and other LEDGINs on HIV-1 expression reported before (8, 36, 38), although the effects seem less pronounced for HIV-2 than for HIV-1.

### GS-9822 retargets HIV-2 integration away from genes and gene-dense regions

The effect of LEDGINs on HIV-1 latency and reactivation is in part due to retargeting of residual integration sites away from gene and gene-dense regions (8, 36, 38). To study if similar changes occur with HIV-2, we transduced 400,000 SupT1 cells with 8.82 × 10^3^ pg of HIV-2 OGH or 2.67 × 10^4^ pg of HIV-2 eGFP for 3 days in the presence of increasing concentrations of GS-9822 (50–400 nM). Cells were then washed and kept in culture for at least 10 days in the absence of the compound to allow for the dilution of non-integrated DNA. Genomic DNA was extracted and prepared for Illumina MiSeq Integration site sequencing. Sequencing data were analyzed using the INSPIIRED platform (58, 59). Addition of GS-9822 resulted in a dose-dependent reduction in the number of copies/cell, up to 15-fold for HIV-2 OGH and 16-fold for HIV-2 eGFP (Fig. S5a). Among the residual integrants, a reduction in the number of unique integration sites was detected for both viruses (up to 9-fold for HIV-2 OGH and 6-fold for HIV-2 eGFP) by the next-generation sequencing analysis (Fig. S5b). This once again confirms that GS-9822 inhibits HIV-2 integration.

We then analyzed the unique integration sites to verify whether GS-9822 retargets residual integrants of HIV-2 OGH and HIV-2 eGFP. We initially chose a range of 50–400 nM of GS-9822 as we saw an increase in the latent fraction on day four at these concentrations, although the effects of GS-9822 on reactivation seemed to require higher concentrations. At these high concentrations, the number of integration sites recovered is too low to perform and correctly interpret statistical analysis. We excluded the 400 nM condition for each vector for the same reason. As fewer unique sites were recovered with HIV-2 OGH, we will focus more on the results of HIV-2 GFP. For each condition, the results are compared with computer-generated matched random controls using a receiver operating characteristic (ROC) curve area (red tile color).

**Fig 5:**
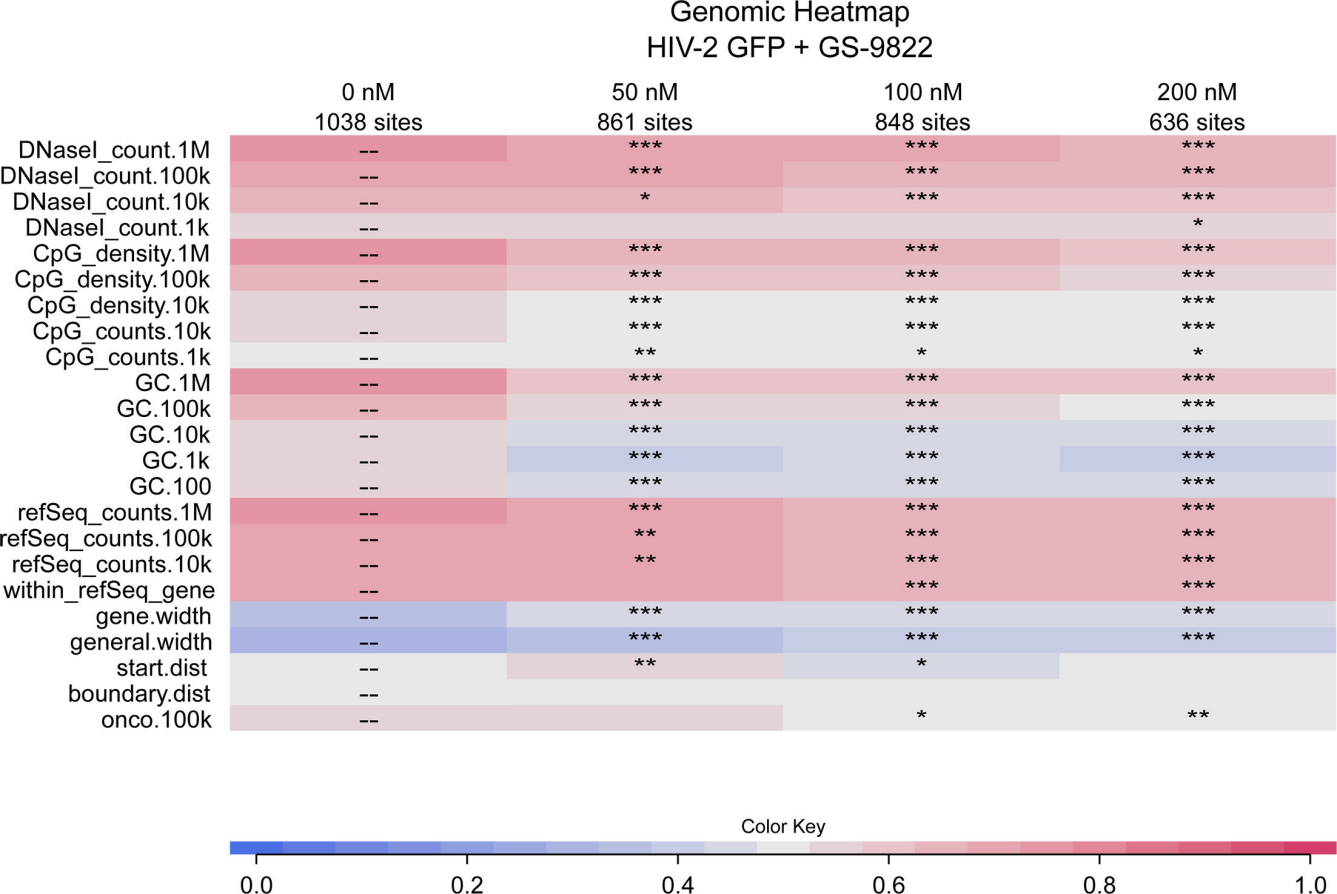
GS-9822 retargets HIV-2 eGFP integration away from gene-dense regions. We transduced 400,000 SupT1 cells with 2.67 × 10^4^ pg of HIV-2 eGFP in the presence or absence of increasing concentrations of GS-9822, kept in culture for at least 10 days. Next, genomic DNA was extracted for Illumina Miseq integration site sequencing, and data were analyzed with the INSPIIRED software and represented as a heat map (58, 59). Colors indicate whether a genomic feature is favored (red) or disfavored (blue) for integration as compared to computer-generated matched random controls (MRCs) using a receiver operating characteristic (ROC) curve area. Each integration site is compared to its MRC, and according to the rank of the integration site, a number is assigned (1 if the feature is favored at the integration site over the MRC, 0 if it is disfavored, and 0.5 if the feature is equal for the two sites). The analysis was done for all integration sites, after which an average was calculated. These ROC curve areas were statistically analyzed using Wald type test statistics which are referred to the χ^2^ distribution (*P < 0.05, **P = 0.01, ***P < 0.0001).

As described previously, HIV-2 strongly favors integration in genes (within_refSeq_gene in table) in the no drug control conditions (Fig. 5; Fig. S6). Upon addition of GS-9822, this preference was reduced for both viruses. This retargeting occurred in a dose-dependent manner for HIV-2 eGFP, although not for HIV-2 OGH, which may be due to the low number of unique sites recovered for HIV-2 OGH overall. However, when compared with matched random controls using a receiver operating characteristic (ROC) curve area, integration in genes was still preferred (red tile color) (Fig. 5; Fig. S6). DNase I hypersensitive sites are a marker for actively transcribed chromatin and are normally preferred by HIV-2 (56). Upon addition of GS-9822, DNase I hypersensitive sites were less frequently found in proximity to residual integrants of HIV-2 eGFP, although once again, integration near DNase I hypersensitive sites was still preferred when compared to matched random controls (red title color). Next, we analyzed a number of markers correlating with the gene density of the regions surrounding the integration sites. First, the RefSeq gene count, measuring the number of genes surrounding these proviruses, decreased as well, across all windows for HIV-2 eGFP and for a window of 1 million base pairs for HIV-2 OGH, while remaining higher than for matched random controls. CpG islands occur most frequently in proximity to promoters, and thus, the CpG count and CpG density correlate with gene density when examined at larger ranges such as windows of 1 million and 100,000 base pairs. HIV-2 favors integration in CpG dense regions at these large ranges, and upon addition of GS-9822, this preference markedly decreased for HIV-2 eGFP and HIV-2 OGH in a dose-dependent manner while still remaining preferred as compared to matched random controls. At a slightly smaller window, such as 10,000 base pairs, the preference of HIV-2 is less pronounced but is reduced to levels similar to the matched random controls. Gene-dense regions also tend to be GC-rich, and GC counts surrounding HIV-2 integrants in the no-drug control were higher than for matched random controls, especially at longer ranges such as 1 million and 100,000 base pairs. Upon GS-9822 treatment, residual integrants were targeted away from GC-rich regions, especially for HIV-2 eGFP, with levels similar to the matched random controls in the 200 nM condition. At smaller ranges, GS-9822 even caused HIV-2 eGFP to integrate less in GC-rich regions compared to matched random controls, although this effect was not concentration dependent. Lastly, gene density is inversely correlated with the width of intergenic regions and genes, and HIV-2 usually favors integration in regions with a lower width of genes and intergenic regions. GS-9822 treatment led to a relative increase in the width of genes and intergenic regions surrounding residual proviruses for HIV-2 eGFP and to a lesser degree for HIV-2 OGH, although the width still remained lower than for matched random controls. Together, these results indicate that the inhibition of the interaction between HIV-2 integrase and LEDGF/p75 by GS-9822 retargets integration away from genes, gene-dense regions, and active chromatin, although HIV-2 still maintained these preferences when compared to matched random controls.

Overall, the shift in HIV-2 integration sites resembles the LEDGIN-mediated retargeting previously demonstrated for HIV-1 (8, 36, 38). To our knowledge, this is the first time an LEDGIN-mediated effect on HIV-2 integration sites is shown.

### Retargeting of HIV-2 integration sites results in a different epigenetic landscape of the residual provirus

**Fig 6:**
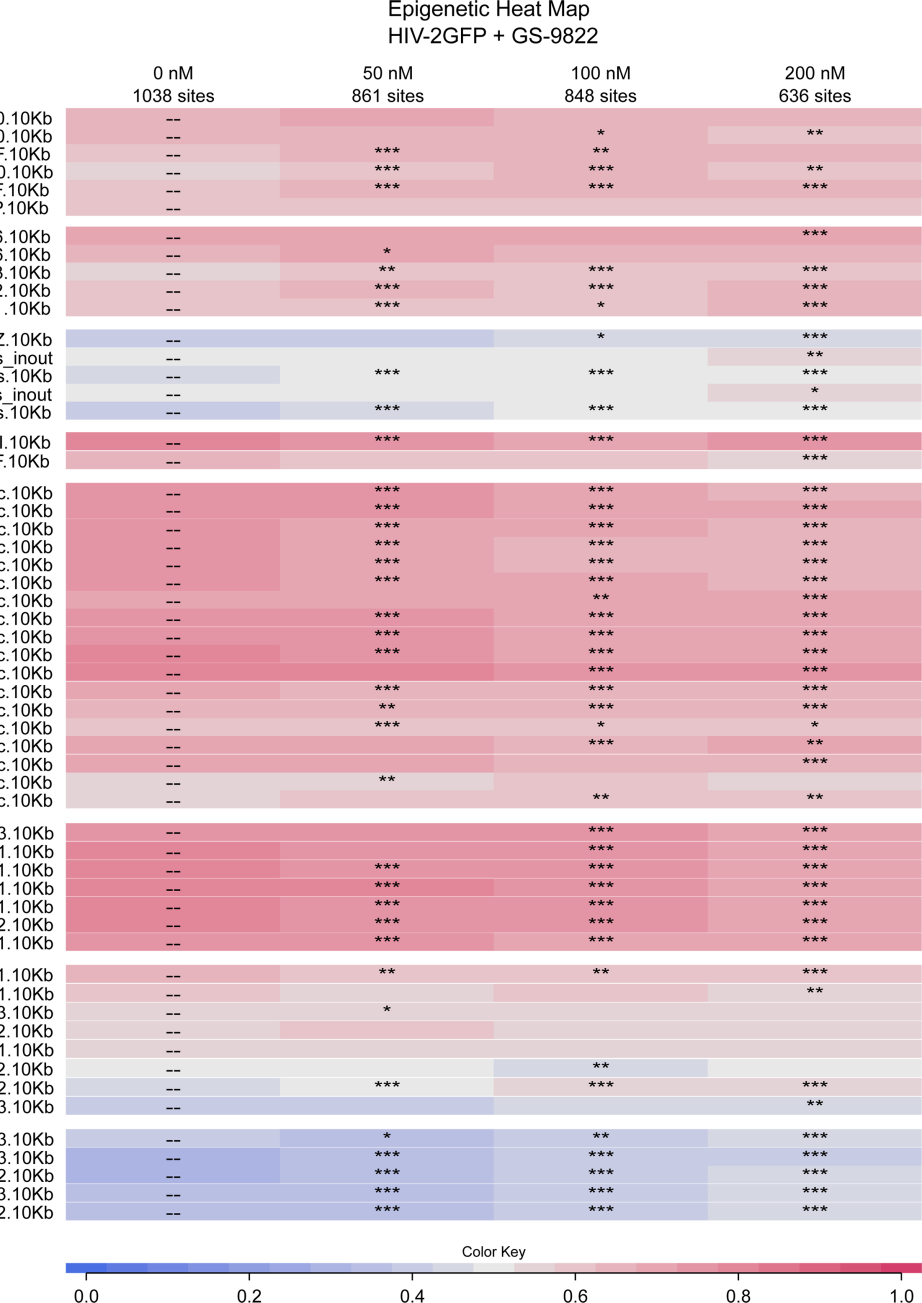
Heat map representation of epigenetic features of HIV-2 eGFP integration after treatment with GS-9822. After Illumina Miseq sequencing, data were analyzed using the INSPIIRED software, yielding the heat map shown (58, 59). Color codes were as described in Fig. 5. The ROC curve areas were statistically analyzed using Wald type test statistics, which are referred to the χ^2^ distribution (*P < 0.05, **P = 0.01, ***P < 0.0001).

Next, we used the INSPIIRED platform to generate an epigenetic heat map to see if LEDGIN treatment affects the epigenetic landscape surrounding residual HIV-2 proviruses, as was demonstrated before for HIV-1 (8, 36, 38) (Fig. 6; Fig. S7). In the no-drug control, HIV-2 preferred integration in actively transcribed regions, when compared to matched random controls, as shown by the preference of both HIV-2 OGH and HIV-2 eGFP for acetylated histone marks (red tile color), including H4K16ac, H4K8ac, and H4K12ac, which are associated with the gene bodies of actively transcribed genes, although the acetylation marks can occur in promoter regions of actively transcribed genes as well (60, 61). Upon the addition of GS-9822, integration was shifted away from these acetylation marks, especially for HIV-2 eGFP, although they remained preferred when compared with matched random controls. Methylated histone marks can be associated with active chromatin, usually when they are mono-methylated. H3K27me1, H2BK5me1, H4K20me1, and H3K9me1 are usually found along active gene bodies (60–67) and are favored by HIV-2 in the no-drug control. HIV-2 likewise favors integration near H3K36me3, the histone marker recognized by LEDGF/p75 (13, 14, 68–70). Addition of GS-9822 shifts integration away from these active methylation marks but does not fully abolish this preference when compared to matched random controls for both HIV-2 OGH and HIV-2 eGFP. Other, mostly bi- or tri-methylated histone marks, like H4K20me3, H3K27me3, H3K27me2, H3K9me3, and H3K9me2, are associated with repressed chromatin (61, 63, 65, 66), and these were disfavored by HIV-2 in the no-drug control (blue tile color). The addition of GS-9822 slightly increased residual integration toward these histone marks, although they were still slightly disfavored when compared to matched random controls. This effect was more pronounced for HIV-2 eGFP than for HIV-2 OGH. HIV-2 integration also showed a preference for integration near Pol II binding sites, which are positively correlated with gene expression levels (61). Upon GS-9822 treatment, integration was shifted away from these sites as well, although this effect was not as pronounced as the effects on other histone markers.

HIV-2 is integrated into regions with fewer nucleosomes and H2AZ. Alike for HIV-1, GS-9822 shifted integrations more toward nucleosome-dense regions, even sometimes resulting in a slight preference for integration there, although this result was not clearly concentration dependent nor for HIV-2 OGH or HIV-2 eGFP. The residual integrants of both HIV-2 OGH and eGFP were slightly shifted toward H2AZ-containing regions, but this effect was less pronounced than for HIV-1 integration sites after GS-9822 treatment (56), and H2AZ remained disfavored for HIV-2 when compared to matched random controls.

Together with the results of the genomic markers, these data show that GS-9822 mediates retargeting of HIV-2 integration away from active transcription units and toward a more repressive epigenetic landscape. As such, GS-9822 treatment results in a shift of HIV-2 integration sites that shows a striking resemblance to the retargeting previously shown for HIV-1 integration sites after LEDGIN treatment (8, 36, 38).

## DISCUSSION

In this study, we show that the new-generation LEDGIN, GS-9822, inhibits the LEDGF/p75-integrase interaction between HIV-2 and SIV integrase. In addition, we report for the first time that a LEDGIN potently inhibits the replication of HIV-2 and SIVmac at nanomolar concentrations in cell culture. During HIV-1 infection, LEDGINs have been shown to inhibit integration and additionally retarget residual integrants away from active transcription units, resulting in a reservoir that is more latent and less prone to reactivation. Using the double reporter construct HIV-2 OGH, we now show for the first time that LEDGIN treatment inhibits HIV-2 integration and that residual proviruses are more latent and less responsive to reactivation. Using integration site sequencing, we confirmed that GS-9822 also retargets HIV-2 integration sites away from active transcription units and toward a more repressive chromatin environment.

GS-9822 inhibited the LEDGF/p75-integrase interaction for HIV-2 and SIV and inhibited the replication of HIV-2 and SIV in cell culture with IC_50_s in the nanomolar range, representing the first report of activity of a LEDGIN against SIV and HIV-2. Previous generations of LEDGINs showed little to no effect against HIV-2 and SIV (23, 27, 44), as the integrase molecules of these viruses only share about 57% sequence identity at the protein level (Fig. S8), and since some amino acids important to the interaction of LEDGF/p75 with HIV-1 integrase are not conserved. For example, A128, A129, and T174 are part of a hydrophobic pocket formed by two HIV-1 integrase monomers, bound by the LEDGF/p75 IBD. In addition, LEDGF/p75 interacts with E170 of the first HIV-1 integrase monomer and W131 of the second HIV-1 integrase monomer, as shown by crystallography and mutagenesis studies (31, 71–73), reviewed in a previous study (74). In contrast, HIV-2 and SIV carry A128M, A129V, and E170N substitutions (Fig. S8). The larger side chain of the methionine at position A128 in particular poses a steric hindrance for LEDGIN binding. One of the ways GS-9822 was optimized against LEDGIN resistance mutations is the addition of an oxetanyl piperidine group, which interacts with W131 and stabilizes the binding of the compound in the case of a T174I mutation (55). As W131 is conserved in HIV-2 and SIV IN, this may stabilize the binding of GS-9822 to HIV-2 and SIV IN as well. Of course, further structural studies or *in silico* modeling would be useful to study the binding of LEDGF/p75 and LEDGINs like GS-9822 with HIV-2 and SIV integrases.

As HIV-2 has remained mostly limited to West Africa and is estimated to have infected only 1–2 million people, research into HIV-2 treatment and latency is scarce (75, 76). Although some antiretrovirals developed for HIV-1, like INSTIs, are also active against HIV-2, many of them (like the non-nucleoside reverse transcriptase inhibitors and some protease inhibitors) are not. If LEDGINs with HIV-2 activity were to be developed, this would add a much-needed option for HIV-2 treatment regimens. Remarkably, patients with HIV-2 often have lower plasma viremia, with 8%–37% of patients naturally controlling the virus and usually experiencing a slower CD4^+^ T-cell decline (77–85). There is little research comparing HIV-1 and HIV-2 latency (86–88), although assuming HIV-2 is more latent than HIV-1 is likely an oversimplification (56). It is tempting to assume that achieving a state of deep latency for HIV-2 may be easier than for HIV-1 because of its less virulent phenotype, but all we can really say is that more research into HIV-2 latency is sorely needed.

In addition to their antiviral effects, LEDGINs have been shown to reduce HIV-1 integration (19–21, 34, 35) and retarget residual proviruses away from active transcription units, resulting in a more repressive epigenetic landscape surrounding the provirus (8, 36, 38). In addition, using a single-round dual reporter virus, HIV-1 OGH (9, 39, 40), residual integrants after LEDGIN treatment were shown to be more latent and more resistant to reactivation (8, 36, 38). We recently developed and characterized a similar double reporter construct for HIV-2, called HIV-2 OGH (56). HIV-2 OGH allows us to distinguish between latently and productively transduced cells and test HIV-2 expression and reactivation. HIV-2 yielded higher reporter gene expression levels than HIV-1 and a lower latent fraction in cell lines. HIV-2 also proved less sensitive to reactivation with TNFα. Here, we used the same HIV-2 OGH construct to test the effect of GS-9822 on HIV-2 latency. Residual, integrated HIV-2 proviruses after GS-9822 treatment displayed reduced gene expression, were more likely to be latent, and were more refractory to reactivation (Fig. 3 and 4). These effects are strikingly similar to those previously reported for HIV-1 after treatment with GS-9822 or other LEDGINs like CX14442 and STP0404 (8, 36, 38, 43). Additionally, we sequenced integration sites of HIV-2 OGH and HIV-2 eGFP in SupT1 cells. As shown before (56), HIV-2 shares the HIV-1 preference for integration in genes and gene-dense regions. We demonstrate for the first time that HIV-2 prefers an active chromatin environment as well (Fig. 5 and 6). In this study, we sequenced HIV-2 integration sites with increasing concentrations of GS-9822 and demonstrated retargeting of residual HIV-2 proviruses away from genes and gene-dense regions and toward a more repressive chromatin environment (Fig. 5 and 6), once again replicating the established effects of LEDGINs on HIV-1 integration (8, 36, 38). As HIV-2 is more similar to SIVmac than to HIV-1, this suggests that LEDGINs like GS-9822 may induce these effects on SIV replication as well, although further research is needed to confirm the latency-inducing effects of GS-9822 on SIV. If confirmed, this would allow researchers to test a “block and lock” strategy using LEDGINs for the first time in primate models with SIV.

The “block-and-lock” functional cure strategy aims to induce a state of deep latency of proviruses within HIV reservoirs. As a result, even without eradicating the virus from the body completely, the viral load will remain suppressed even in the absence of ART. Due to the complex and diverse nature of HIV latent reservoirs, it is likely that a single latency-promoting agent (LPA) will not suffice to silence each provirus. Indeed, previous research with LEDGINs and HIV-1 has shown proviruses with high viral expression even after treatment with high doses of LEDGINs (8). Recently, our lab showed that the combination of ZL0580 and LEDGIN CX14442 leads to a near-complete block of HIV-1 expression at a cellular level (89), while other combinations of LPAs, like LEDGINS plus didehydrocortistatin A (90), and the effects of these combinations on HIV-2 remain to be investigated. It will probably also be beneficial to combine different cure strategies. For example, combining enhanced immune control achieved by broadly neutralizing antibodies with more latent reservoirs caused by LPAs and elimination of proviruses through gene therapy. Our study does have some limitations. First, while we focused on the effects of LEDGINs on integration and integration sites (early effects of LEDGINs) for this paper, LEDGINs affect multiple stages of the HIV viral life cycle. LEDGINs promote integrase multimerization during assembly, resulting in crippled progeny virions with the viral RNA genome displaced outside the capsid core (27–30). This late effect of LEDGINs significantly contributes to antiviral potency and tends to occur at lower concentrations than the early effects of LEDGINs, although the difference between the two concentrations can vary between compounds. For example, the late effect occurred at 3-fold lower concentrations for CX05045 (27), while STP0404 shows less effect at the early stage, despite inhibiting LEDGF/p75-integrase binding (43). Our study focused mainly on the early effect of LEDGINs, as this is the most relevant for the latency-promoting effects of LEDGINs and their potential use in a block-and-lock functional cure strategy. Therefore, we did not separately investigate the late effect here, although we did study the effect of GS-9822 in wild-type infection, which includes both the early and late effects of LEDGINs. Although different experimental set-ups do not allow for direct comparison, GS-9822 inhibited HIV-1 integration with an IC_50_ of 6.9 nM when using HIV-1 OGH in SupT1 cells and inhibited multiple round infection in MT4 cells with an EC_50_ of 2 nM (38). For HIV-2, we observed an inhibition of integration with an IC_50_ of 33.5 nM, while the inhibition of multiple round infection has an EC_50_ of 305 nM. The fact that LEDGINs have a multimodal mechanism of action speaks to their versatility. Indeed, as we now show that LEDGINs can be developed to target HIV-2 and SIV, researchers may be able to develop more potent compounds with increased multimerization or late effects, or compounds with a more pronounced effect on the early effect.

Additionally, we did not sequence integrated proviruses to rule out mutations in the mKO2 promoter, which could hamper mKO2 expression and confound the results. However, since our experiments involve replication-deficient double reporter viruses in a single round experiment, mutations are unlikely to occur. Moreover, LEDGINs shift the nuclear topology of HIV-1 integration away from the nuclear periphery and more toward the center of the nucleus (36), which we did not investigate for HIV-2. Next, the latency-promoting effects of GS-9822 on reactivation seemed to occur mostly at high concentrations of about 1,000 nM, although we did see an increase in the latent fraction on day 4 at lower concentrations of about 100 nM. Unfortunately, we were not able to test concentrations above 200 nM in our integration site sequencing analysis due to low numbers of recovered integration sites at these concentrations, as LEDGINs inhibit integration as well. It is therefore possible that our data still underestimate the effects LEDGINs have on the retargeting of HIV-2 integration.

Finally, further development of GS-9822 is hampered due to toxicity observed in a cynomolgus monkey model (55). A reproducible, dose-dependent vacuolation of the urothelium of the kidney, bladder, and ureter was observed through pathological evaluation, with additional elevations in urinary proteins, red blood cells, and urobilinogen at the highest dose tested (55). This toxicity was not observed in rats, and the lesions seemed to be restricted to the monkey urothelium (91). Recently, the results of further investigation into the mechanism for this toxicity were published, and the authors suspect physicochemical changes of GS-9822 due to differences in bladder pH. Indeed, GS-9822 forms a bilayer at the air/water interface at pH 5, which is highly disruptive to the cell membrane due to a zwitterionic characteristic of the molecule (91). As we worked mainly with T-cell lines in our experiments and the toxicity was shown in urothelial cells only, we did not perform extensive toxicity assays, although our MTT/MT4 assay did include the calculation of a CC_50_ value. The differential structures of other advanced LEDGINs, such as BI 224436 and STP0404, suggest that a similar toxic effect of these LEDGINs is unlikely. Indeed, toxicology studies with BI 224,436 (tested in rats, monkeys, and dogs [32]) and STP0404 (tested in rats and dogs [43]) did not show urothelial vacuolation; hence, this toxicity is probably not a class effect. Unfortunately, it has already been shown that the A128T mutation confers resistance to both BI 224436 and STP0404 and that these LEDGINs are ineffective against SIV (32, 43). Still, as our study proves that some LEDGINs can indeed inhibit HIV-2 and SIV replication, potent LEDGINs against these viruses should be developed and tested, as animal model testing of the effects of LEDGINs on latency will be necessary to investigate the potential of LEDGINs not just as antivirals, but as part of a “block-and-lock” functional cure strategy.

In summary, we provide the first evidence of the antiviral activity of LEDGINs against HIV-2 and SIV replication using the LEDGIN congener GS-9822. GS-9822 inhibited the LEDGF/p75-integrase interaction of HIV-2 and SIV integrase and retargeted HIV-2 integration away from active genes and toward a more repressive chromatin environment. In addition, GS-9822 treatment resulted in residual HIV-2 integrants that were more latent and less prone to reactivation, thus recapitulating all aspects of the early effect of LEDGINs previously established for HIV-1. Although the development of GS-9822 has been halted due to toxicity in cynomolgus monkeys, our study shows (i) the potential to develop LEDGINs that inhibit HIV-2 and SIV replication, and (ii) the confirmation of the effect of LEDGINs, including retargeting and latency enhancement for HIV-2.

## MATERIALS AND METHODS

### Compounds

CX14442 (33) was synthesized at Cistim/CD3 KU Leuven (courtesy of Dr. A. Marchand). GS-9822 (55) was provided by Gilead (courtesy of M. Balakrishnan). All compounds were diluted in DMSO to a stock concentration of 25 mM, which was then diluted in a 1/10 series and aliquoted for use in the different experiments.

### Cell culture

All cells were tested to be mycoplasma-free (PlasmoTest, InvivoGen) and cultured in a humidified atmosphere at 5% CO2 and 37°C. SupT1 cells were provided by the National Institutes of Health (NIH) AIDS reagent program. They were cultured in Roswell Park Memorial Institute 1640 (RPMI, GIBCO) supplemented with 10% vol/vol fetal bovine serum (FBS, GIBCO) and 0.01% vol/vol gentamicin (GIBCO). HEK293T cells were purchased from ATCC (293T/17 [HEK 293T/17] (ATCC CRL-11268)) and cultured in Dulbecco’s modified Eagle medium (DMEM, GIBCO) with 5% FBS (GIBCO) and 0.01% gentamicin (GIBCO).

### Reporter viruses

#### HIV-2 eGFP

HIV-2 eGFP is a replication-deficient reporter virus based on HIV-2 ROD (ENA/EMBL Accession number X05291) with an LTR-driven enhanced Green Fluorescent Protein (eGFP) in the *nef* position. This reporter virus was a kind gift from T. Hatziioannou (Aaron Diamond AIDS Research Center, place, USA).

#### HIV-1 OGH

The dual-colored reporter virus, HIV-1 OGH, is an orange-green variant of a previously described LAI-based double reporter virus (9, 39, 40) used to study HIV latency (9, 36, 38, 92). It is replication-deficient and contains an LTR-driven enhanced Green Fluorescent Protein (eGFP) in the *nef* position, followed by a constitutively active EF1α promoter driving a mutant Kusabira-Orange 2 (mKO2) reporter. The double reporter virus was a kind gift from the Verdin lab (Buck Institute for Research on Aging, California, USA).

#### pNL4.3 OGH

The pNL4-3.Luc.RE- was also used to generate a pNL4.3 OGH plasmid in a similar fashion, which was later used as the basis for HIV-2 OGH. pNL4.3-Luc.RE- was cut with *NotI* and *XhoI* to delete the *luciferase* gene insert. Next, a gene block containing the OGH-cassette was ordered from Sigma (Paulien) (sequence in supplemental material), amplified using primers OGH_NotI_Fw AAAAAAAGCGGCCGCTGTCTCC and OGH_XhoI_Rv TTTTTTTCTCGAGCTAGCTGTAGTGGGC, cut with *NotI* and *XhoI,* and cloned into the previously digested pNL4.3 backbone. The resulting plasmid was sequenced to confirm correct insertion.

#### HIV-2 OGH

HIV-2 OGH was cloned by our lab based on HIV-1 OGH (see above). Both HIV-1 and HIV-2 OGH are replication-deficient and contain a LTR-driven enhanced Green Fluorescent Protein (eGFP) in the *nef* position, followed by a constitutively active EF1α promoter driving a mutant Kusabira-Orange 2 (mKO2) reporter.

To generate HIV-2 OGH, we cloned the OGH cassette into the HIV-2 eGFP backbone. Our lab had previously cloned a pNL4.3 OGH plasmid, based on pNL4-3.Luc.RE, which was obtained through the NIH AIDS Research and Reagent Reference program. pNL4.3-Luc.RE- was cut with *NotI* and *XhoI* to delete the *luciferase* gene insert. Next, a gene block containing the OGH-cassette was ordered from Sigma (sequence in supplemental material), amplified using primers OGH_NotI_Fw AAAAAAAGCGGCCGCTGTCTCC and OGH_XhoI_Rv TTTTTTTCTCGAGCTAGCTGTAGTGGGC, cut with *NotI* and *XhoI* and cloned into the previously digested pNL4.3 backbone. The resulting plasmid was sequenced to confirm correct insertion. We used pNL4.3OGH to create HIV-2 OGH. First, we inserted a *NotI* restriction site before the eGFP cassette in HIV-2 eGFP with Q5 site-directed mutagenesis (New England Biolabs) using primers HIV-2_NotI_forward ccgcATGGTGAGCAAGGGCGAG and HIV-2_NotI_reverse ccgcATTGCAAGAAGGCTGTTCTAAG. After sequencing to confirm correct insertion, this plasmid was digested with *NotI* and *BspeI* (located downstream of the eGFP cassette of HIV-2 eGFP) to remove the eGFP cassette. Next, we needed to adapt the OGH cassette for insertion into the HIV-2 backbone. We started by changing the *XhoI* restriction site in pNL4.3OGH to a *BspeI* site using Q5 site-directed mutagenesis (New England Biolabs) with primers pNL4.3OGH_BspeI_Fw CTACAGCTAGtccggaACCTAGAAAAACATGGAGCAATCACAAGTAGC and pNL4.3OGH_BspeI_Rv TGGGCCACGGCGTCTTCC. The resulting OGH cassette flanked by *NotI* and *BspeI* was next amplified with primers OGH_NotI_Fw AAAAAAAGCGGCCGCTGTCTCC and OGH_BspeI_rv GCCCACTACAGCTAGTCCGGAAAAAAAA. The resulting amplicon was digested with *NotI* and *BspeI* and cloned into the similarly digested HIV-2 backbone. Since the OGH cassette did not contain a start codon before eGFP, although this was the case for the eGFP in HIV-2 eGFP, a start codon was re-inserted with Q5 site-directed mutagenesis using primers HIV2atgOGH_fw GATGGCGGCCGCTGTCTCCAAA and HIV2atgOGH_rv TTAAGATTGCAAGAAGGCTGTTCTAAGTCTC. The resulting plasmid was sequenced for correct insertion. Upon transduction of SupT1 with HIV-2 OGH, we noticed a lower mKO2 median fluorescence intensity (data not shown) compared to HIV-1 OGH. Upon re-analyzing the sequences of the original OGH cassette and the OGH gene block from pNL4.3 OGH, we discovered that to shorten the gene block, a Kozak sequence between EF1a and mKO2 had been removed. Therefore, we cloned this sequence into HIV-2 OGH using Q5 site-directed mutagenesis (New England Biolabs) with primers HIV2OGH_Kozak_Fw CGGTCGCCACCATGGTTTCTGTGATCAAGCCCGAAATG and HIV2OGH_Kozak_Rv GTAGCGCTAGCGTAGGCGCCGGTCACAGC. The resulting plasmid was once again sequenced to confirm correct insertion. Upon transduction, mKO2 expression levels matched HIV-1 OGH (data not shown).

### Virus production

HIV-2 eGFP and OGH viruses were produced in HEK293T cells by double transfection with pHIV-2 eGFP, pHIV-2 OGH plasmids, respectively, and pVSVG, the VSV-G protein encoding plasmid, using linear polyethylenimine (PEI, Polysciences) in Optimem (GIBCO). First, HEK293T cells were seeded overnight in DMEM (GIBCO) at 5.7 million cells per Petri dish. A DNA mixture was made consisting of 20 µg transfer plasmid and 5 µg pVSVG diluted in 700 µL of Optimem per Petri dish. Next, a PEI mixture containing 68 µL of 10 µM PEI in 632 µL of 150 mM NaCl was added gently to the plasmid mixture. After incubating for 15 min at room temperature, 5 mL of Optimem was added, and the resulting medium was used to replace the seeding medium. After 6 h, the plates were washed twice with phosphate-buffered saline (PBS), and the medium was replaced with Optimem. After 72 h, the supernatant was collected for the first time, and the medium was replaced for a second collection after another 24 h. The supernatant was filtered through a 0.45-µm pore membrane (Merck), concentrated using a Vivaspin with a 15–50 kDa cutoff (Merck), treated with 100 U/mL DNase (Roche Diagnostics) for 1 h at 37°C, and stored at −80°C until use.

### Viral strains

All wild-type viral strains were obtained through the NIH AIDS Reagent Program, Division of AIDS, NIAID, NIH. The origin of the different virus strains is as follows: HIV-1 III_B_ (93–95), HIV-1 NL4-3 (96), HIV-2 EHO virus (97), HIV-2 ROD virus (98), and SIV MAC251 (99).

### Expression and purification of recombinant proteins

His_6_-tagged HIV-1 (strain NL4-3), HIV-2 (strain ROD), and SIV (strain MAC251) integrase and Flag-tagged LEDGF/p75 were purified for AlphaScreen tests as described previously, over a Hi-Trap nickel affinity gel (Ni^2+^-NTA) and HiTrap heparin column, respectively (100, 101).

### AlphaScreen assays

We performed LEDGF/p75-integrase interaction AlphaScreen assays (Perkin Elmer, Waltham, MA) as described previously (23). The reaction buffer contained 25 mM Tris/HCl (pH 7.3), 150 mM NaCl, 1 mM MgCl_2_, 0.1% Tween 20, 0.1% Bovine Serum Albumin (BSA). All compounds and proteins were diluted to a 5× working solution in this buffer. We used a final volume of 25 µL in a 384-well microtiter plate (OptiPlate^TM^-384, Perkin Elmer). We used purified His_6_-tagged integrase (His-IN) protein from HIV-1, HIV-2, or SIV, recognized by a Nickel-chelate donor bead and Flag-tagged LEDGF/p75 (Flag-p75), recognized by an anti-FLAG-coated acceptor bead. First, AlphaScreen assays without compounds were run, with a range of concentrations (final concentrations 10–200 µM) for both proteins to determine the optimal reaction conditions (data not shown); 5 µL of His-IN was incubated for 1 h with 5 µL of Flag-LEDGF/p75 at 4°C, and 10 µL of a mixture of nickel-chelate donor beads and anti-FLAG-coated acceptor beads at a 1/100 dilution was added. The plate was then incubated for 3 h at 30°C, while limiting exposure of the reaction to light. The emission of light from the acceptor beads was analyzed in the Envision plate reader (Perkin Elmer) in AlphaScreen mode. We confirmed the specificity of the assay by competing Flag-LEDGF/p75 with unlabeled LEDGF/p75. Increasing concentrations of unlabeled LEDGF p75 (0–1,000 nM) were incubated with 50 nM of his-tagged integrase of HIV-1 and HIV-2 for 30 min. Next, 100 nM Flag-tagged LEDGF p75 was added to the mixture for 1 h. Finally, a 1/100 dilution of His-donor beads and Flag-acceptor beads was added to the mixture. The experiment was performed two times with one technical replicate per experiment. Background signal from wells containing beads only (no protein) was subtracted. At 1,000 nM, untagged LEDGF/p75 reduced the AlphaScreen signal by 83% for HIV-1 and 91% for HIV-2 (data not shown).

Next, we determined dose-response curves in the presence of the compounds; 5 µl of His-IN at a final concentration of 50 nM was first incubated for 30 min at 4°C in the presence of 5 µL of varying concentrations of the compounds (final concentrations of 0.1–500 µM for CX14442 and 0.001–500 µM of GS-9822). Next, 5 µL of Flag-tagged LEDGF/p75 at a final concentration of 100 nM was added for an additional 1 h at 4°C. Further steps were performed as described above. Data were analyzed using the Envision Manager Software (Perkin Elmer) and GraphPad Prism.

### MTT assay

In this assay, a yellow-colored dye (3-(4,5-dimethylthiazol-2-yl)−2,5-diphenyltetrazolium bromide (MTT) is reduced by a mitochondrial dehydrogenase in metabolically active cells to a purple formazan derivative (102), which is measured spectrophotometrically. Here, we used the MTT assay to determine the inhibition of HIV-induced cytopathic effects (CPE) in MT-4 cells, and thus, the inhibition of HIV-1 infection. MT-4 cells were infected with wild-type HIV-1 subtype B viruses (III_B_ and NL4.3), HIV-2 ROD (subtype A), HIV-2 EHO (subtype B), and SIV MAC at a multiplicity of infection (MOI) of 0.01 in the presence of a dilution series of CX14442 and GS-9822 (1.28 nM to 100 µM for CX14442 and 0.064 nM to 5 µM for GS-9822). The MTT assay was performed after 5 days.

### Multiple round replication p24 assay

MT-4 cells were infected with wild-type HIV-1 IIIb (subtype B) virus, HIV-2 ROD (subtype A) at an MOI of 0.0001, and SIV MAC at an MOI of 0.000,005 in the presence of 0.05 and 1 µM of GS-9822. After 1 day, the infecting virus and compound were washed away. Cells were kept in culture until day 9, and the supernatant was collected every day for analysis with p24 ELISA as per the manufacturer’s instructions (Fujirebio Europe).

### Transduction and reactivation experiments

In total, 100,000 SupT1 or cells were transduced with 2.76 × 10^3^ to 4.41 × 10^3^ pg of HIV-2 OGH or 5.55 × 10^3^ to 1.52 × 10^4^ pg of HIV-2 eGFP, for 3 days in the presence of GS-9822 (55), after which the cells were washed twice with PBS. Flow cytometry analysis was performed on days 3 (data not shown) and day 4. Nine to 10 days post-transduction, half the cells were reactivated in duplicate with 10 ng/mL Tumor Necrosis Factor α (TNFα, Immunosource), while the other half were left untreated. For one additional experiment, reactivation was performed on day 8. Flow cytometry analysis was performed 24 and 48 h post-reactivation, without any additional reactivation. Drug concentrations are listed for each individual experiment. IC_50_ values were calculated using GraphPad Prism (GraphPad Software).

### Flow cytometry

Cells were fixed in 2% paraformaldehyde (PFA) for 15 min at room temperature and stored at 4°C. Cells transduced with HIV-2 eGFP were analyzed with a Guava Easycyte 5HT flow cytometer (Merck, Overijse, Belgium) with a 488 nm, 50 mW laser, and 525/30 nm band pass filters in two experiments. Cells transduced with HIV-2 OGH, and cells transduced with HIV-2 eGFP in three other experiments, were washed with and resuspended in PBS before flow cytometry analysis. For the HIV-1 and HIV-2 OGH constructs, cells were analyzed with a MACS Quant VYB analyzer (Mylteny Biotech). eGFP expression was measured using a 488 nm, 50 mW DPSS (diode-pumped solid-state) excitation laser, and the emitted signal was measured after a 525/50 nm band pass filter. mKO2 expression was measured using a 561 nM, 100 mW diode excitation laser, and a 586/15 nm band pass filter. The gating strategy involved gating for lymphocytes using the forward and side scatter channels (FSC-H/SSC-H) and exclusion of doublets (FSC-A/FSC-H). At least 15,000 single live cells were counted in total, and each sample was measured in duplicate. All data were analyzed using FlowJo software (FlowJo LLC) and plotted using GraphPad Prism software.

### Proviral copies/cell qPCR analysis

#### HIV-2 Gag qPCR

HIV-2 copies were quantified with a real-time Gag qPCR adapted from Bertine et. al., 2017 (103). The PCR mix contained 12.5 µL IQ Supermix (Biorad), 0.25 µL of 2 primers each at 20 µM concentration (HIV-2_Fw GCGCGAGAAACTCCGTCTTG and HIV-2_Rv TTCGCTGCCCACACAATATGTT), 1 µL of probe (5′-FAM-CAGGTTACGGCCCGGCGGAAAGA-TAM), 6 µL of water, and 5 µL of lysed cells. The PCR reaction started with 2 min at 50°C and 10 min at 95°C, followed by 50 cycles of 95°C for 30 s and 60°C for 1 min.

#### CCR5 qPCR

To normalize for total input DNA, a CCR5 qPCR was performed on the same samples, as previously described (104). The PCR mix contained 5 µL of genomic DNA, 10 µL of Sybr Green (Invitrogen), 1 µL of 2 primers each at 20 µM concentration (LK46: GCTGTGTTTGCGTCTCTCCCAGGA; LK47: CTCACAGCCCTGTGCCTCTTCTTC), and 3 µL of water. The PCR reaction was performed in a LightCycler 480 (Roche Life Science). All samples were run at least in duplicate. Data were analyzed using the provided LightCycler 480 software.

### Integration site analysis

Integration sites were determined as previously described (58). SupT1 cells were seeded and transduced with HIV-2 OGH or HIV-2 eGFP for 3 days. After 3 days, the cells were washed twice with PBS and kept in culture for at least 10 days to allow non-integrated DNA to be diluted. Next, genomic DNA was extracted using the GenElute Mammalian Genomic DNA miniprep kit (Sigma-Aldrich) and sheared by sonication with the Covaris M220. Linkers were added to the sheared DNA ends, and integration sites were amplified by nested PCR with primers complementary to the linker and the viral LTR. Finally, the PCR products were sequenced by Illumina Miseq (paired-end, 300 cycles). Sequencing data were analyzed using INSPIRED software (59).

## Data Availability

All relevant data are within the manuscript and its Supporting information files. HIV-2 integration sites with annotation, as obtained through analysis with the INSPIIRED software (59), are provided as Supplemental material. In addition, we submitted demultiplexed raw sequencing data for each sample to NCBI Sequence Read Archive under theBioProject accession number: PRJNA1320943. The original multiplexed sequencing data may be obtained by submitting a motivated request to the corresponding author.

## SUPPLEMENTAL MATERIAL

Data S1 spectrum.01699-25-s0001.xlsx HIV-2 GFP genomic integration sites.

Data S2 spectrum.01699-25-s0002.xlsb Epigenetic integration sites HIV-2 GFP.

Data S3 spectrum.01699-25-s0003.xlsx Genomic integration sites HIV-2 OGH.

Data S4 spectrum.01699-25-s0004.xlsb Epigenetic integration sites HIV-2 OGH.

Supplemental figures spectrum.01699-25-s0005.docx Fig. S1 to S8.

ASM does not own the copyrights to Supplemental Material that may be linked to, or accessed through, an article. The authors have granted ASM a non-exclusive, world-wide license to publish the Supplemental Material files. Please contact the corresponding author directly for reuse.

## References

[1] Dahabieh MS, Battivelli E, Verdin E. 2015. Understanding HIV latency: the road to an HIV cure. Annu Rev Med 66:407–421. doi:10.1146/annurev-med-092112-152941 25587657 PMC4381961

[2] Blankson JN, Persaud D, Siliciano RF, In PR, Ecycling PAR. 2002. The challenge of viral reservoirs in HIV-1 infection. Annu Rev Med 53:557–593. doi:10.1146/annurev.med.53.082901.104024 11818490

[3] Symons J, Cameron PU, Lewin SR. 2018. HIV integration sites and implications for maintenance of the reservoir. Curr Opin HIV AIDS 13:152–159. doi:10.1097/COH.0000000000000438 29206656 PMC5808998

[4] Deeks SG, Archin N, Cannon P, Collins S, Jones RB, de Jong MAWP, Lambotte O, Lamplough R, Ndung’u T, Sugarman J, Tiemessen CT, Vandekerckhove L, Lewin SR, International AIDS Society (IAS) Global Scientific Strategy working group. 2021. Research priorities for an HIV cure: international AIDS society global scientific strategy 2021. Nat Med 27:2085–2098. doi:10.1038/s41591-021-01590-5 34848888

[5] Jordan A, Bisgrove D, Verdin E. 2003. HIV reproducibly establishes a latent infection after acute infection of T cells in vitro. EMBO J 22:1868–1877. doi:10.1093/emboj/cdg188 12682019 PMC154479

[6] Lewinski MK, Bisgrove D, Shinn P, Chen H, Hoffmann C, Hannenhalli S, Verdin E, Berry CC, Ecker JR, Bushman FD. 2005. Genome-wide analysis of chromosomal features repressing human immunodeficiency virus transcription. J Virol 79:6610–6619. doi:10.1128/JVI.79.11.6610-6619.2005 15890899 PMC1112149

[7] Chen H-C, Martinez JP, Zorita E, Meyerhans A, Filion GJ. 2017. Position effects influence HIV latency reversal. Nat Struct Mol Biol 24:47–54. doi:10.1038/nsmb.3328 27870832

[8] Vansant G, Chen H-C, Zorita E, Trejbalová K, Miklík D, Filion G, Debyser Z. 2020. The chromatin landscape at the HIV-1 provirus integration site determines viral expression. Nucleic Acids Res 48:7801–7817. doi:10.1093/nar/gkaa536 32597987 PMC7641320

[9] Battivelli E, Dahabieh MS, Abdel-Mohsen M, Svensson JP, Tojal Da Silva I, Cohn LB, Gramatica A, Deeks S, Greene WC, Pillai SK, Verdin E. 2018. Distinct chromatin functional states correlate with HIV latency reactivation in infected primary CD4^+^ T cells. eLife 7:e34655. doi:10.7554/eLife.34655 29714165 PMC5973828

[10] Schröder ARW, Shinn P, Chen H, Berry C, Ecker JR, Bushman F. 2002. HIV-1 integration in the human genome favors active genes and local hotspots. Cell 110:521–529. doi:10.1016/S0092-8674(02)00864-4 12202041

[11] Lewinski MK, Yamashita M, Emerman M, Ciuffi A, Marshall H, Crawford G, Collins F, Shinn P, Leipzig J, Hannenhalli S, Berry CC, Ecker JR, Bushman FD. 2006. Retroviral DNA integration: viral and cellular determinants of target-site selection. PLoS Pathog 2:e60. doi:10.1371/journal.ppat.0020060 16789841 PMC1480600

[12] Brady T, Agosto LM, Malani N, Berry CC, O’Doherty U, Bushman F. 2009. HIV integration site distributions in resting and activated CD4^+^ T cells infected in culture. AIDS 23:1461–1471. doi:10.1097/QAD.0b013e32832caf28 19550285 PMC2862484

[13] van Nuland R, van Schaik FM, Simonis M, van Heesch S, Cuppen E, Boelens R, Timmers HM, van Ingen H. 2013. Nucleosomal DNA binding drives the recognition of H3K36-methylated nucleosomes by the PSIP1-PWWP domain. Epigenetics Chromatin 6:12. doi:10.1186/1756-8935-6-12 23656834 PMC3663649

[14] Eidahl JO, Crowe BL, North JA, McKee CJ, Shkriabai N, Feng L, Plumb M, Graham RL, Gorelick RJ, Hess S, Poirier MG, Foster MP, Kvaratskhelia M. 2013. Structural basis for high-affinity binding of LEDGF PWWP to mononucleosomes. Nucleic Acids Res 41:3924–3936. doi:10.1093/nar/gkt074 23396443 PMC3616739

[15] Llano M, Vanegas M, Hutchins N, Thompson D, Delgado S, Poeschla EM. 2006. Identification and characterization of the chromatin-binding domains of the HIV-1 integrase interactor LEDGF/p75. J Mol Biol 360:760–773. doi:10.1016/j.jmb.2006.04.073 16793062

[16] Cherepanov P, Maertens G, Proost P, Devreese B, Van Beeumen J, Engelborghs Y, De Clercq E, Debyser Z. 2003. HIV-1 integrase forms stable tetramers and associates with LEDGF/p75 protein in human cells. J Biol Chem 278:372–381. doi:10.1074/jbc.M209278200 12407101

[17] Ortiz-Hernandez GL, Sanchez-Hernandez ES, Casiano CA. 2020. Twenty years of research on the DFS70/LEDGF autoantibody-autoantigen system: many lessons learned but still many questions. Autoimmun Highlights 11. doi:10.1186/s13317-020-0126-4 PMC7065333 32127038

[18] Cherepanov P, Devroe E, Silver PA, Engelman A. 2004. Identification of an evolutionarily conserved domain in human lens epithelium-derived growth factor/transcriptional co-activator p75 (LEDGF/p75) that binds HIV-1 integrase. J Biol Chem 279:48883–48892. doi:10.1074/jbc.M406307200 15371438

[19] Ciuffi A, Llano M, Poeschla E, Hoffmann C, Leipzig J, Shinn P, Ecker JR, Bushman F. 2005. A role for LEDGF/p75 in targeting HIV DNA integration. Nat Med 11:1287–1289. doi:10.1038/nm1329 16311605

[20] Shun M-C, Raghavendra NK, Vandegraaff N, Daigle JE, Hughes S, Kellam P, Cherepanov P, Engelman A. 2007. LEDGF/p75 functions downstream from preintegration complex formation to effect gene-specific HIV-1 integration. Genes Dev 21:1767–1778. doi:10.1101/gad.1565107 17639082 PMC1920171

[21] Marshall HM, Ronen K, Berry C, Llano M, Sutherland H, Saenz D, Bickmore W, Poeschla E, Bushman FD. 2007. Role of PSIP1/LEDGF/p75 in lentiviral infectivity and integration targeting. PLoS One 2:e1340. doi:10.1371/journal.pone.0001340 18092005 PMC2129110

[22] De Rijck J, Bartholomeeusen K, Ceulemans H, Debyser Z, Gijsbers R. 2010. High-resolution profiling of the LEDGF/p75 chromatin interaction in the ENCODE region. Nucleic Acids Res 38:6135–6147. doi:10.1093/nar/gkq410 20484370 PMC2952859

[23] Christ F, Voet A, Marchand A, Nicolet S, Desimmie BA, Marchand D, Bardiot D, Van der Veken NJ, Van Remoortel B, Strelkov SV, De Maeyer M, Chaltin P, Debyser Z. 2010. Rational design of small-molecule inhibitors of the LEDGF/p75-integrase interaction and HIV replication. Nat Chem Biol 6:442–448. doi:10.1038/nchembio.370 20473303

[24] Demeulemeester J, Chaltin P, Marchand A, De Maeyer M, Debyser Z, Christ F. 2014. LEDGINs, non-catalytic site inhibitors of HIV-1 integrase: a patent review (2006 - 2014). Expert Opin Ther Pat 24:609–632. doi:10.1517/13543776.2014.898753 24666332

[25] Choi E, Mallareddy JR, Lu D, Kolluru S. 2018. Recent advances in the discovery of small-molecule inhibitors of HIV-1 integrase. Future Sci OA 4. doi:10.4155/fsoa-2018-0060 PMC6222271 30416746

[26] Engelman AN. 2019. Multifaceted HIV integrase functionalities and therapeutic strategies for their inhibition. J Biol Chem 294:15137–15157. doi:10.1074/jbc.REV119.006901 31467082 PMC6791320

[27] Desimmie BA, Schrijvers R, Demeulemeester J, Borrenberghs D, Weydert C, Thys W, Vets S, Van Remoortel B, Hofkens J, De Rijck J, Hendrix J, Bannert N, Gijsbers R, Christ F, Debyser Z. 2013. LEDGINs inhibit late stage HIV-1 replication by modulating integrase multimerization in the virions. Retrovirology (Auckl) 10:57. doi:10.1186/1742-4690-10-57 PMC3671127 23721378

[28] Jurado KA, Wang H, Slaughter A, Feng L, Kessl JJ, Koh Y, Wang W, Ballandras-Colas A, Patel PA, Fuchs JR, Kvaratskhelia M, Engelman A. 2013. Allosteric integrase inhibitor potency is determined through the inhibition of HIV-1 particle maturation. Proc Natl Acad Sci USA 110:8690–8695. doi:10.1073/pnas.1300703110 23610442 PMC3666754

[29] Balakrishnan M, Yant SR, Tsai L, O’Sullivan C, Bam RA, Tsai A, Niedziela-Majka A, Stray KM, Sakowicz R, Cihlar T. 2013. Non-catalytic site HIV-1 integrase inhibitors disrupt core maturation and induce a reverse transcription block in target cells. PLoS One 8:e74163. doi:10.1371/journal.pone.0074163 24040198 PMC3767657

[30] Le Rouzic E, Bonnard D, Chasset S, Bruneau J-M, Chevreuil F, Le Strat F, Nguyen J, Beauvoir R, Amadori C, Brias J, Vomscheid S, Eiler S, Lévy N, Delelis O, Deprez E, Saïb A, Zamborlini A, Emiliani S, Ruff M, Ledoussal B, Moreau F, Benarous R. 2013. Dual inhibition of HIV-1 replication by integrase-LEDGF allosteric inhibitors is predominant at the post-integration stage. Retrovirology (Auckl) 10:144. doi:10.1186/1742-4690-10-144 PMC4222603 24261564

[31] Cherepanov P, Sun ZYJ, Rahman S, Maertens G, Wagner G, Engelman A. 2005. Solution structure of the HIV-1 integrase-binding domain in LEDGF/p75. Nat Struct Mol Biol 12:526–532. doi:10.1038/nsmb937 15895093

[32] Fenwick C, Amad M, Bailey MD, Bethell R, Bös M, Bonneau P, Cordingley M, Coulombe R, Duan J, Edwards P, et al.. 2014. Preclinical profile of BI 224436, a novel HIV-1 non-catalytic-site integrase inhibitor. Antimicrob Agents Chemother 58:3233–3244. doi:10.1128/AAC.02719-13 24663024 PMC4068430

[33] Christ F, Shaw S, Demeulemeester J, Desimmie BA, Marchand A, Butler S, Smets W, Chaltin P, Westby M, Debyser Z, Pickford C, Marchan A, Butler S, Smets W, Chaltin P, Westby M, Debyser Z, Pickford C. 2012. Small-molecule inhibitors of the LEDGF/p75 binding site of integrase block HIV replication and modulate integrase multimerization. Antimicrob Agents Chemother 56:4365–4374. doi:10.1128/AAC.00717-12 22664975 PMC3421592

[34] Schrijvers R, De Rijck J, Demeulemeester J, Adachi N, Vets S, Ronen K, Christ F, Bushman FD, Debyser Z, Gijsbers R. 2012. LEDGF/p75-independent HIV-1 replication demonstrates a role for HRP-2 and remains sensitive to inhibition by LEDGINs. PLoS Pathog 8:e1002558. doi:10.1371/journal.ppat.1002558 22396646 PMC3291655

[35] Schrijvers R, Vets S, De Rijck J, Malani N, Bushman FD, Debyser Z, Gijsbers R. 2012. HRP-2 determines HIV-1 integration site selection in LEDGF/p75 depleted cells. Retrovirology (Auckl) 9:84. doi:10.1186/1742-4690-9-84 PMC3485173 23046603

[36] Vranckx LS, Demeulemeester J, Saleh S, Boll A, Vansant G, Schrijvers R, Weydert C, Battivelli E, Verdin E, Cereseto A, Christ F, Gijsbers R, Debyser Z. 2016. LEDGIN-mediated Inhibition of Integrase-LEDGF/p75 interaction reduces reactivation of residual latent HIV. EBioMedicine 8:248–264. doi:10.1016/j.ebiom.2016.04.039 27428435 PMC4919729

[37] Feng L, Dharmarajan V, Serrao E, Hoyte A, Larue RC, Slaughter A, Sharma A, Plumb MR, Kessl JJ, Fuchs JR, Bushman FD, Engelman AN, Griffin PR, Kvaratskhelia M. 2016. The competitive interplay between allosteric HIV-1 integrase inhibitor BI/D and LEDGF/p75 during the early stage of HIV-1 replication adversely affects inhibitor potency. ACS Chem Biol 11:1313–1321. doi:10.1021/acschembio.6b00167 26910179 PMC4874862

[38] Bruggemans A, Vansant G, Balakrishnan M, Mitchell ML, Cai R, Christ F, Debyser Z. 2023. GS-9822, a preclinical LEDGIN candidate, displays a block-and-lock phenotype in cell culture. Antimicrob Agents Chemother 65:e02328-20. doi:10.1128/AAC.02328-20 33619061 PMC8092873

[39] Chavez L, Calvanese V, Verdin E. 2015. HIV latency is established directly and early in both resting and activated primary CD4 T cells. PLoS Pathog 11:e1004955. doi:10.1371/journal.ppat.1004955 26067822 PMC4466167

[40] Calvanese V, Chavez L, Laurent T, Ding S, Verdin E. 2013. Dual-color HIV reporters trace a population of latently infected cells and enable their purification. Virology (Auckl) 446:283–292. doi:10.1016/j.virol.2013.07.037 PMC4019006 24074592

[41] Abrahams MR, Joseph SB, Garrett N, Tyers L, Moeser M, Archin N, Council OD, Matten D, Zhou S, Doolabh D, Anthony C, Goonetilleke N, Karim SA, Margolis DM, Pond SK, Williamson C, Swanstrom R. 2019. The replication-competent HIV-1 latent reservoir is primarily established near the time of therapy initiation. Sci Transl Med 11. doi:10.1126/scitranslmed.aaw5589 PMC7233356 31597754

[42] Aslanyan S, Ballow CH, Sabo JP, Habeck J, Roos D, MacGregor TR, Robinson P, Kort J. 2011. Safety and pharmacokinetics (PK) of single rising oral doses of a novel HIV integrase inhibitor in healthy volunteers. ICAAC

[43] Maehigashi T, Ahn S, Kim U-I, Lindenberger J, Oo A, Koneru PC, Mahboubi B, Engelman AN, Kvaratskhelia M, Kim K, Kim B. 2021. A highly potent and safe pyrrolopyridine-based allosteric HIV-1 integrase inhibitor targeting host LEDGF/p75-integrase interaction site. PLoS Pathog 17:e1009671. doi:10.1371/journal.ppat.1009671 34293041 PMC8297771

[44] Amadori C, van der Velden YU, Bonnard D, Orlov I, van Bel N, Le Rouzic E, Miralles L, Brias J, Chevreuil F, Spehner D, Chasset S, Ledoussal B, Mayr L, Moreau F, García F, Gatell J, Zamborlini A, Emiliani S, Ruff M, Klaholz BP, Moog C, Berkhout B, Plana M, Benarous R. 2017. The HIV-1 integrase-LEDGF allosteric inhibitor MUT-A: resistance profile, impairment of virus maturation and infectivity but without influence on RNA packaging or virus immunoreactivity. Retrovirology (Auckl) 14:50. doi:10.1186/s12977-017-0373-2 PMC5680779 29121950

[45] Desimmie BA, Demeulemeester J, Christ F, Debyser Z. 2013. Rational design of LEDGINs as first allosteric integrase inhibitors for the treatment of HIV infection. Drug Discov Today Technol 10:e517–e522. doi:10.1016/j.ddtec.2012.10.002 24451643

[46] Busschots K, Vercammen J, Emiliani S, Benarous R, Engelborghs Y, Christ F, Debyser Z. 2005. The interaction of LEDGF/p75 with integrase is lentivirus-specific and promotes DNA binding. J Biol Chem 280:17841–17847. doi:10.1074/jbc.M411681200 15749713

[47] Cherepanov P. 2007. LEDGF/p75 interacts with divergent lentiviral integrases and modulates their enzymatic activity in vitro. Nucleic Acids Res 35:113–124. doi:10.1093/nar/gkl885 17158150 PMC1802576

[48] MacNeil A, Sankalé J-L, Meloni ST, Sarr AD, Mboup S, Kanki P. 2006. Genomic sites of human immunodeficiency virus type 2 (HIV-2) integration: similarities to HIV-1 in vitro and possible differences in vivo. J Virol 80:7316–7321. doi:10.1128/JVI.00604-06 16840312 PMC1563694

[49] Crise B, Li Y, Yuan C, Morcock DR, Whitby D, Munroe DJ, Arthur LO, Wu X. 2005. Simian immunodeficiency virus integration preference is similar to that of human immunodeficiency virus type 1. J Virol 79:12199–12204. doi:10.1128/JVI.79.19.12199-12204.2005 16160146 PMC1211548

[50] Hematti P, Hong B-K, Ferguson C, Adler R, Hanawa H, Sellers S, Holt IE, Eckfeldt CE, Sharma Y, Schmidt M, von Kalle C, Persons DA, Billings EM, Verfaillie CM, Nienhuis AW, Wolfsberg TG, Dunbar CE, Calmels B. 2004. Distinct genomic integration of MLV and SIV vectors in primate hematopoietic stem and progenitor cells. PLoS Biol 2:e423. doi:10.1371/journal.pbio.0020423 15550989 PMC529319

[51] Wang GP, Ciuffi A, Leipzig J, Berry CC, Bushman FD. 2007. HIV integration site selection: analysis by massively parallel pyrosequencing reveals association with epigenetic modifications. Genome Res 17:1186–1194. doi:10.1101/gr.6286907 17545577 PMC1933515

[52] Mitchell RS, Beitzel BF, Schroder ARW, Shinn P, Chen H, Berry CC, Ecker JR, Bushman FD. 2004. Retroviral DNA integration: ASLV, HIV, and MLV show distinct target site preferences. PLoS Biol 2:E234. doi:10.1371/journal.pbio.0020234 15314653 PMC509299

[53] Soto MJ, Peña A, Vallejo FG. 2011. A genomic and bioinformatics analysis of the integration of HIV in peripheral blood mononuclear cells. AIDS Res Hum Retroviruses 27:547–555. doi:10.1089/AID.2010.0182 20919923

[54] Monse H, Laufs S, Kuate S, Zeller WJ, Fruehauf S, Uberla K. 2006. Viral determinants of integration site preferences of simian immunodeficiency virus-based vectors. J Virol 80:8145–8150. doi:10.1128/JVI.00373-06 16873270 PMC1563816

[55] Mitchell ML, Balakrishnan M, Brizgys G, Cai R, Landson E, Mulato A, Osier M, Wang J, Yu H. 2017. Novel non-catalytic site integrase inhibitor with improved resistance profile. CROI Conference. Seattle, Washington

[56] Bruggemans A, Vansant G, Van de Velde P, Debyser Z. 2023. The HIV-2 OGH double reporter virus shows that HIV-2 is less cytotoxic and less sensitive to reactivation from latency than HIV-1 in cell culture. J Virus Erad 9:100343. doi:10.1016/j.jve.2023.100343 37701289 PMC10493508

[57] Dahabieh MS, Ooms M, Brumme C, Taylor J, Harrigan PR, Simon V, Sadowski I. 2014. Direct non-productive HIV-1 infection in a T-cell line is driven by cellular activation state and NFκB. Retrovirology (Auckl) 11:17. doi:10.1186/1742-4690-11-17 PMC4015675 24502247

[58] Sherman E, Nobles C, Berry CC, Six E, Wu Y, Dryga A, Malani N, Male F, Reddy S, Bailey A, Bittinger K, Everett JK, Caccavelli L, Drake MJ, Bates P, Hacein-Bey-Abina S, Cavazzana M, Bushman FD. 2017. INSPIIRED: a pipeline for quantitative analysis of sites of new DNA integration in cellular genomes. Mol Ther Methods Clin Dev 4:39–49. doi:10.1016/j.omtm.2016.11.002 28344990 PMC5363316

[59] Berry CC, Nobles C, Six E, Wu Y, Malani N, Sherman E, Dryga A, Everett JK, Male F, Bailey A, Bittinger K, Drake MJ, Caccavelli L, Bates P, Hacein-Bey-Abina S, Cavazzana M, Bushman FD. 2017. INSPIIRED: quantification and visualization tools for analyzing integration site distributions. Mol Ther Methods Clin Dev 4:17–26. doi:10.1016/j.omtm.2016.11.003 28344988 PMC5363318

[60] Wang Z, Zang C, Rosenfeld JA, Schones DE, Barski A, Cuddapah S, Cui K, Roh TY, Peng W, Zhang MQ, Zhao K. 2008. Combinatorial patterns of histone acetylations and methylations in the human genome. Nat Genet 40:897–903. doi:10.1038/ng.154 18552846 PMC2769248

[61] Barski A, Cuddapah S, Cui K, Roh T-Y, Schones DE, Wang Z, Wei G, Chepelev I, Zhao K. 2007. High-resolution profiling of histone methylations in the human genome. Cell 129:823–837. doi:10.1016/j.cell.2007.05.009 17512414

[62] Bannister AJ, Schneider R, Myers FA, Thorne AW, Crane-Robinson C, Kouzarides T. 2005. Spatial distribution of di- and tri-methyl lysine 36 of histone H3 at active genes. J Biol Chem 280:17732–17736. doi:10.1074/jbc.M500796200 15760899

[63] Berger SL. 2007. The complex language of chromatin regulation during transcription. Nature 447:407–412. doi:10.1038/nature05915 17522673

[64] Hon GC, Hawkins RD, Ren B. 2009. Predictive chromatin signatures in the mammalian genome. Hum Mol Genet 18:R195–201. doi:10.1093/hmg/ddp409 19808796 PMC2912651

[65] Kimura H. 2013. Histone modifications for human epigenome analysis. J Hum Genet 58:439–445. doi:10.1038/jhg.2013.66 23739122

[66] Mellor J, Dudek P, Clynes D. 2008. A glimpse into the epigenetic landscape of gene regulation. Curr Opin Genet Dev 18:116–122. doi:10.1016/j.gde.2007.12.005 18295475

[67] Huff JT, Plocik AM, Guthrie C, Yamamoto KR. 2010. Reciprocal intronic and exonic histone modification regions in humans. Nat Struct Mol Biol 17:1495–1499. doi:10.1038/nsmb.1924 21057525 PMC3057557

[68] Debyser Z, Christ F, De Rijck J, Gijsbers R. 2015. Host factors for retroviral integration site selection. Trends Biochem Sci 40:108–116. doi:10.1016/j.tibs.2014.12.001 25555456

[69] Pradeepa MM, Sutherland HG, Ule J, Grimes GR, Bickmore WA. 2012. Psip1/Ledgf p52 binds methylated histone H3K36 and splicing factors and contributes to the regulation of alternative splicing. PLoS Genet 8:e1002717. doi:10.1371/journal.pgen.1002717 22615581 PMC3355077

[70] Marini B, Kertesz-Farkas A, Ali H, Lucic B, Lisek K, Manganaro L, Pongor S, Luzzati R, Recchia A, Mavilio F, Giacca M, Lusic M. 2015. Nuclear architecture dictates HIV-1 integration site selection. Nature 521:227–231. doi:10.1038/nature14226 25731161

[71] Busschots K, Voet A, De Maeyer M, Rain J-C, Emiliani S, Benarous R, Desender L, Debyser Z, Christ F. 2007. Identification of the LEDGF/p75 binding site in HIV-1 integrase. J Mol Biol 365:1480–1492. doi:10.1016/j.jmb.2006.10.094 17137594

[72] Emiliani S, Mousnier A, Busschots K, Maroun M, Van Maele B, Tempé D, Vandekerckhove L, Moisant F, Ben-Slama L, Witvrouw M, Christ F, Rain J-C, Dargemont C, Debyser Z, Benarous R. 2005. Integrase mutants defective for interaction with LEDGF/p75 are impaired in chromosome tethering and HIV-1 replication. J Biol Chem 280:25517–25523. doi:10.1074/jbc.M501378200 15855167

[73] Hombrouck A, De Rijck J, Hendrix J, Vandekerckhove L, Voet A, De Maeyer M, Witvrouw M, Engelborghs Y, Christ F, Gijsbers R, Debyser Z. 2007. Virus evolution reveals an exclusive role for LEDGF/p75 in chromosomal tethering of HIV. PLoS Pathog 3:e47. doi:10.1371/journal.ppat.0030047 17397262 PMC1839165

[74] Christ F, Debyser Z. 2013. The LEDGF/p75 integrase interaction, a novel target for anti-HIV therapy. Virology (Auckl) 435:102–109. doi:10.1016/j.virol.2012.09.033 23217620

[75] Gottlieb GS, Raugi DN, Smith RA. 2018. 90-90-90 for HIV-2? Ending the HIV-2 epidemic by enhancing care and clinical management of patients infected with HIV-2. Lancet HIV 5:e390–e399. doi:10.1016/S2352-3018(18)30094-8 30052509

[76] Joint United Nations Programme on HIV/AIDS (UNAIDS). 2023. World AIDS Day 2023 UNAIDS Fact Sheet

[77] van der Loeff MFS, Larke N, Kaye S, Berry N, Ariyoshi K, Alabi A, van Tienen C, Leligdowicz A, Sarge-Njie R, da Silva Z, Jaye A, Ricard D, Vincent T, Jones SR, Aaby P, Jaffar S, Whittle H. 2010. Undetectable plasma viral load predicts normal survival in HIV-2-infected people in a West African village. Retrovirology (Auckl) 7. doi:10.1186/1742-4690-7-46 PMC2887382 20482865

[78] Thiébaut R, Matheron S, Taieb A, Brun-Vezinet F, Chêne G, Autran B, immunology group of the ANRS CO5 HIV-2 cohort. 2011. Long-term nonprogressors and elite controllers in the ANRS CO5 HIV-2 cohort. AIDS 25:865–867. doi:10.1097/QAD.0b013e328344892e 21358376

[79] Gottlieb GS, Sow PS, Hawes SE, Ndoye I, Redman M, Coll-Seck AM, Faye-Niang MA, Diop A, Kuypers JM, Critchlow CW, Respess R, Mullins JI, Kiviat NB. 2002. Equal plasma viral loads predict a similar rate of CD4^+^ T cell decline in human immunodeficiency virus (HIV) type 1- and HIV-2-infected individuals from Senegal, West Africa. J Infect Dis 185:905–914. doi:10.1086/339295 11920314

[80] Popper SJ, Sarr AD, Guèye-Ndiaye A, Mboup S, Essex ME, Kanki PJ. 2000. Low plasma human immunodeficiency virus type 2 viral load is independent of proviral load: low virus production in vivo. J Virol 74:1554–1557. doi:10.1128/jvi.74.3.1554-1557.2000 10627569 PMC111493

[81] Marlink R, Kanki P, Thior I, Travers K, Eisen G, Siby T, Traore I, Hsieh CC, Dia MC, Gueye EH, Hellinger J, Guèye-Ndiaye A, Sankalé JL, Ndoye I, Mboup S, Essex M. 1994. Reduced rate of disease development after HIV-2 infection as compared to HIV-1. Science 265:1587–1590. doi:10.1126/science.7915856 7915856

[82] MacNeil A, Sarr AD, Sankalé J-L, Meloni ST, Mboup S, Kanki P. 2007. Direct evidence of lower viral replication rates in vivo in human immunodeficiency virus type 2 (HIV-2) infection than in HIV-1 infection. J Virol 81:5325–5330. doi:10.1128/JVI.02625-06 17329334 PMC1900238

[83] Andersson S, Norrgren H, da Silva Z, Biague A, Bamba S, Kwok S, Christopherson C, Biberfeld G, Albert J. 2000. Plasma viral load in HIV-1 and HIV-2 singly and dually infected individuals in Guinea-Bissau, West Africa: significantly lower plasma virus set point in HIV-2 infection than in HIV-1 infection. Arch Intern Med 160:3286–3293. doi:10.1001/archinte.160.21.3286 11088091

[84] Esbjörnsson J, Månsson F, Kvist A, da Silva ZJ, Andersson S, Fenyö EM, Isberg P-E, Biague AJ, Lindman J, Palm AA, Rowland-Jones SL, Jansson M, Medstrand P, Norrgren H, Sweden and Guinea-Bissau Cohort Research Group. 2018. Long-term follow-up of HIV-2-related AIDS and mortality in Guinea-Bissau: a prospective open cohort study. Lancet HIV:S2352-3018(18)30254-6. doi:10.1016/S2352-3018(18)30254-6 30392769

[85] Soares RS, Tendeiro R, Foxall RB, Baptista AP, Cavaleiro R, Gomes P, Camacho R, Valadas E, Doroana M, Lucas M, Antunes F, Victorino RMM, Sousa AE. 2011. Cell-associated viral burden provides evidence of ongoing viral replication in aviremic HIV-2-infected patients. J Virol 85:2429–2438. doi:10.1128/JVI.01921-10 21159859 PMC3067805

[86] Saleh S, Vranckx L, Gijsbers R, Christ F, Debyser Z. 2017. Insight into HIV-2 latency may disclose strategies for a cure for HIV-1 infection. J Virus Erad 3:7–14. doi:10.1016/S2055-6640(20)30300-9 28275453 PMC5337426

[87] Vidya Vijayan KK, Karthigeyan KP, Tripathi SP, Hanna LE. 2017. Pathophysiology of CD4+ T-Cell depletion in HIV-1 and HIV-2 infections. Front Immunol 8:580. doi:10.3389/fimmu.2017.00580 28588579 PMC5440548

[88] Esbjörnsson J, Jansson M, Jespersen S, Månsson F, Hønge BL, Lindman J, Medina C, da Silva ZJ, Norrgren H, Medstrand P, Rowland-Jones SL, Wejse C. 2019. HIV-2 as a model to identify a functional HIV cure. AIDS Res Ther 16:24. doi:10.1186/s12981-019-0239-x 31484562 PMC6727498

[89] Pellaers E, Janssens J, Wils L, Denis A, Bhat A, Van Belle S, Feng D, Christ F, Zhan P, Debyser Z. 2025. BRD4 modulator ZL0580 and LEDGINs additively block and lock HIV-1 transcription. Nat Commun 16:4226. doi:10.1038/s41467-025-59398-7 40335477 PMC12059001

[90] Mousseau G, Kessing CF, Fromentin R, Trautmann L, Chomont N, Valente ST. 2015. The tat inhibitor didehydro-cortistatin A prevents HIV-1 reactivation from latency. mBio 6:e00465. doi:10.1128/mBio.00465-15 26152583 PMC4495168

[91] Roberts RA, Campbell RA, Sikakana P, Sadler C, Osier M, Xu Y, Feng JY, Mitchell M, Sakowicz R, Chester A, Paoli E, Wang J, Burns-Naas LA. 2021. Species-specific urothelial toxicity with an anti-HIV noncatalytic site integrase inhibitor (NCINI) is related to unusual pH-dependent physicochemical changes. Toxicol Sci 183:105–116. doi:10.1093/toxsci/kfab073 34117767

[92] Vansant G, Vranckx LS, Zurnic I, Van Looveren D, Van de Velde P, Nobles C, Gijsbers R, Christ F, Debyser Z. 2019. Impact of LEDGIN treatment during virus production on residual HIV-1 transcription. Retrovirology (Auckl) 16:8. doi:10.1186/s12977-019-0472-3 PMC6444612 30940165

[93] Popovic M, Sarngadharan MG, Read E, Gallo RC. 1984. Detection, isolation, and continuous production of cytopathic retroviruses (HTLV-III) from patients with AIDS and pre-AIDS. Science 224:497–500. doi:10.1126/science.6200935 6200935

[94] Popovic M, Read-Connole E, Gallo RC. 1984. T4 positive human neoplastic cell lines susceptible to and permissive for HTLV-III. Lancet 2:1472–1473. doi:10.1016/s0140-6736(84)91666-0 6151082

[95] Ratner L, Haseltine W, Patarca R, Livak KJ, Starcich B, Josephs SF, Doran ER, Rafalski JA, Whitehorn EA, Baumeister K, Ivanoff L, Petteway SR Jr, Pearson ML, Lautenberger JA, Papas TS, Ghrayeb J, Chang NT, Gallo RC, Wong-Staal F. 1985. Complete nucleotide sequence of the AIDS virus, HTLV-III. Nature 313:277–284. doi:10.1038/313277a0 2578615

[96] Adachi A, Gendelman HE, Koenig S, Folks T, Willey R, Rabson A, Martin MA. 1986. Production of acquired immunodeficiency syndrome-associated retrovirus in human and nonhuman cells transfected with an infectious molecular clone. J Virol 59:284–291. doi:10.1128/JVI.59.2.284-291.1986 3016298 PMC253077

[97] Clavel F, Guyader M, Guétard D, Sallé M, Montagnier L, Alizon M. 1986. Molecular cloning and polymorphism of the human immune deficiency virus type 2. Nature 324:691–695. doi:10.1038/324691a0 3025743

[98] Rey MA, Krust B, Laurent AG, Guétard D, Montagnier L, Hovanessian AG. 1989. Characterization of an HIV-2-related virus with a smaller sized extracellular envelope glycoprotein. Virology (Auckl) 173:258–267. doi:10.1016/0042-6822(89)90242-0 2683362

[99] Franchini G, Gurgo C, Guo HG, Gallo RC, Collalti E, Fargnoli KA, Hall LF, Wong-Staal F, Reitz MS. 1987. Sequence of simian immunodeficiency virus and its relationship to the human immunodeficiency viruses. Nature 328:539–543. doi:10.1038/328539a0 3497350

[100] Maertens G, Cherepanov P, Pluymers W, Busschots K, De Clercq E, Debyser Z, Engelborghs Y. 2003. LEDGF/p75 is essential for nuclear and chromosomal targeting of HIV-1 integrase in human cells. J Biol Chem 278:33528–33539. doi:10.1074/jbc.M303594200 12796494

[101] Bartholomeeusen K, De Rijck J, Busschots K, Desender L, Gijsbers R, Emiliani S, Benarous R, Debyser Z, Christ F. 2007. Differential interaction of HIV-1 integrase and JPO2 with the C terminus of LEDGF/p75. J Mol Biol 372:407–421. doi:10.1016/j.jmb.2007.06.090 17669426

[102] Pauwels R, Balzarini J, Baba M, Snoeck R, Schols D, Herdewijn P, Desmyter J, De Clercq E. 1988. Rapid and automated tetrazolium-based colorimetric assay for the detection of anti-HIV compounds. J Virol Methods 20:309–321. doi:10.1016/0166-0934(88)90134-6 2460479

[103] Bertine M, Gueudin M, Mélard A, Damond F, Descamps D, Matheron S, Collin F, Rouzioux C, Plantier JC, Avettand-Fenoel V. 2017. New highly sensitive real-time PCR assay for HIV-2 group A and group B DNA quantification. J Clin Microbiol 55:2850–2857. doi:10.1128/JCM.00755-17 28701422 PMC5648720

[104] Zhang L, Lewin SR, Markowitz M, Lin H-H, Skulsky E, Karanicolas R, He Y, Jin X, Tuttleton S, Vesanen M, Spiegel H, Kost R, van Lunzen J, Stellbrink H-J, Wolinsky S, Borkowsky W, Palumbo P, Kostrikis LG, Ho DD. 1999. Measuring recent thymic emigrants in blood of normal and HIV-1–Infected Individuals before and after effective therapy. J Exp Med 190:725–732. doi:10.1084/jem.190.5.725 10477556 PMC2195623

