# Supplementary material for: The LEDGIN GS-9822 inhibits HIV-2 infection and enhances HIV-2 latency
Source: Microbiol Spectr. 2026 Apr 27;14(6):e01699-25. doi: 10.1128/spectrum.01699-25 (PMC13228034; doi:10.1128/spectrum.01699-25)
Supplement: Supplemental figures — Fig. S1 to S8. [file spectrum.01699-25-s0005.docx]

The LEDGIN GS-9822 inhibits HIV-2 infection and enhances HIV-2 latency.

**Anne Bruggemans**, Gerlinde Vansant, Paulien Van de Velde, Frauke Christ, Zeger Debyser

#### Supplementary figure 1


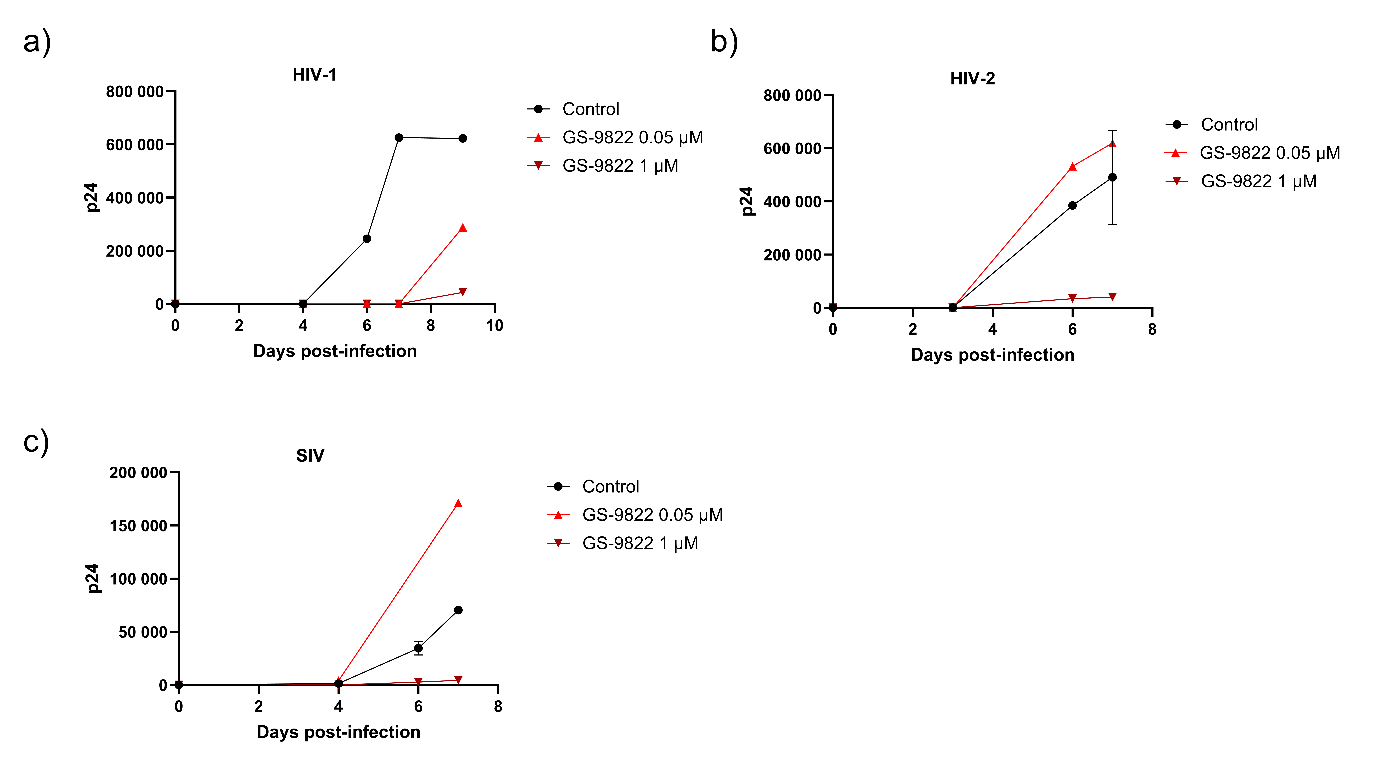


Figure I Treatment with GS-9822 inhibits HIV-1, HIV-2 and SIV replication.
MT4-cells were infected with wild-type viruses in the presence of 0.05 and 1µM of GS-9822. After 1 day, the infecting virus and compound were washed away. Cells were kept in culture until day 9 and supernatant was collected every day for analysis with p24 ELISA. a) HIV-1 IIIb (subtype B) virus, b) HIV-2 ROD (subtype A) virus, c) SIV MAC virus.

#### Supplementary figure 2


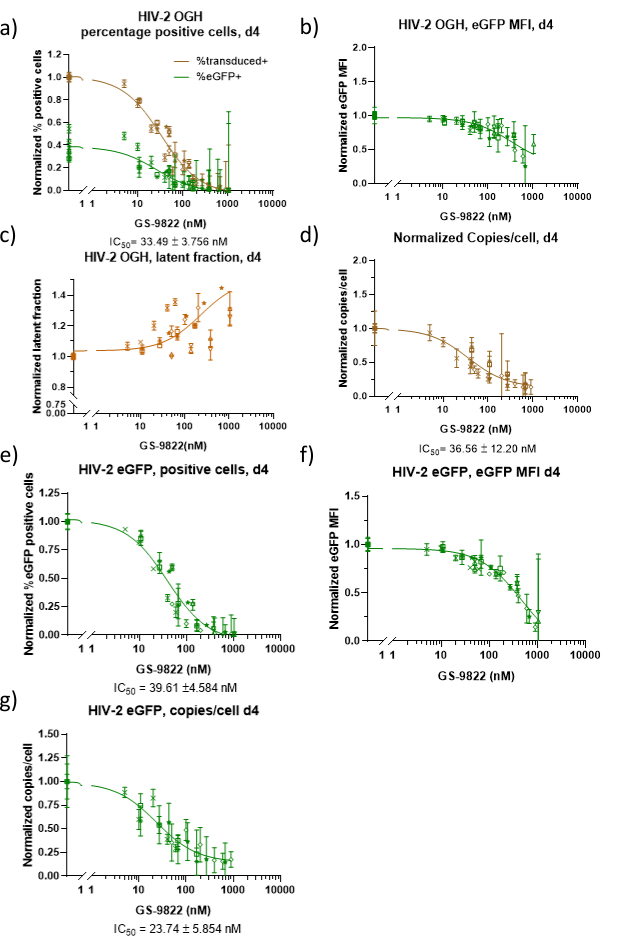


Figure II Treatment with GS-9822 inhibits transduction, decreases expression and increases immediate latency.
a-c) Summary of 7 experiments where 1 x 10^5^ SupT1 cells were transduced with 3.30-4.41 x 10^3^ pg of HIV-2 OGH in the presence of increasing concentrations of GS-9822, normalized to the value for the no-drug control. Each data point represents the mean and SD from one experiment, with one color per experiment. a) Percentage of eGFP^+^ and transduced^+^ cells on day 4 post-transduction. Both the percentage of transduced and positive cells and the percentage of eGFP^+^ cells were normalized to the percentage of transduced+ cells in the no-drug control. The IC_50_ with SD for the decline in transduced positive cells is listed below, b) eGFP MFI, c) Latent fraction, calculated as previously described. d) Copies/cell, calculated as previously described. Summary data of 5 experiments. Each data point represents the mean and SD from one, experiment with one color per experiment. The IC_50_ with SD is listed below, e-g) Summary data measured on day 4 of 7 experiments where 1x10^5^ SupT1 cells with 1.33 -1.78 x10^4^ pg HIV-2 eGFP or GS-9822, normalized to the value for the no-drug control. Each data point represents mean and SD from one experiment, with one color per experiment. e) Percentage of eGFP positive cells on day 4 post-transduction. The IC_50_ with SD for the decline in eGFP positive cells is listed below, f) eGFP MFI. g) Copies/cell, calculated as previously described. Summary data of 5 experiments. Each data point represents the mean and SD from one, experiment with one color per experiment. The IC_50_ with SD is listed below.

#### Supplementary figure 3


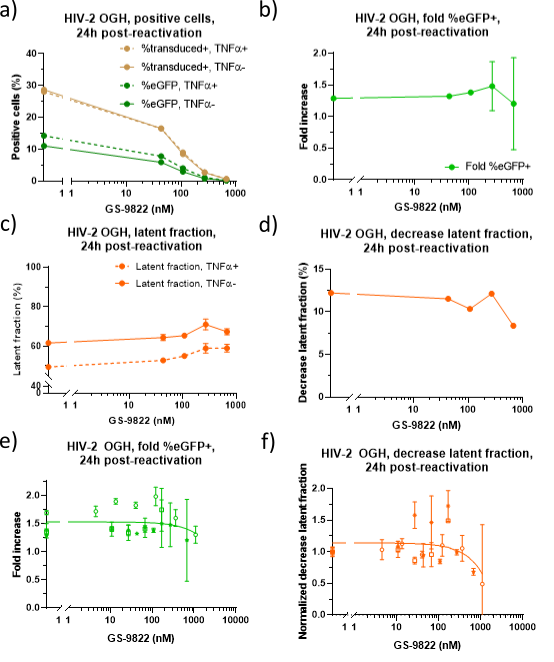


Figure III Treatment with GS-9822 decreases reactivation from latency at 24 h.
a-d) Flow cytometry data on day 11 or 24 hrs after reactivation with 10 ng/ml TNFα of SupT1 cells previously transduced with 2.48 x 10^4^ pg of HIV-2 OGH. Cells were pre-treated with increasing concentrations of GS-9822. Full lines represent non-activated cells, dotted lines represent TNFα treated cells. Data show one representative experiment out of 4 and the averages of duplicate measurements with standard deviation are plotted. a) Percentage of transduced positive (eGFP or mKO2 positive cells) and eGFP positive cells, b) Fold increase in eGFP positive cells (%eGFP positive TNFα treated cells/ %eGFP positive untreated cells), c)The latent fraction, (percentage of single mKO2 positive cells/(percentage of transduced positive cells)*100) or (quadrant C/ (quadrant A+B+C)) as shown in Fig. 2c in TNFα treated and untreated cells, d) Upon reactivation, the latent fraction decreases and the plotted decrease in latent fraction is calculated by subtracting the latent fraction in the TNFα treated condition from the latent fraction in the non-treated condition, e, f) Summary data of 4 experiments, were 1x10^5^ SupT1 cells were transduced with 2.76- 4.40 x10^3^ pg of HIV-2 OGH and pre-treated with GS-9822. Each data point represents the mean and SD from one experiment, with one color per experiment. Transduction was done in the same way for all experiments, but for 1 experiment reactivation was performed on day 8 instead of day 10, e) Fold increase in eGFP positive cells, calculated as previously described, f) Decrease in latent fraction, calculated as previously described. All values were normalized to the decrease in latent fraction in the no-drug control.

#### Supplementary figure 4


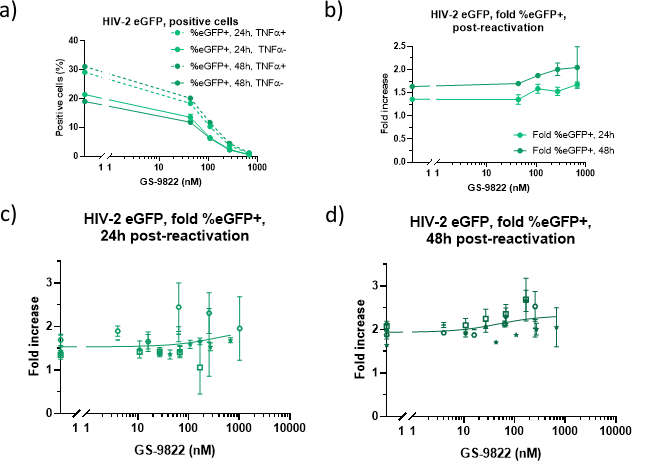


Figure IV Effect of GS-9822 pre-treatment on expression after reactivation of HIV-2 eGFP transduced cells.
a-b) Flow cytometry data 24 hrs or 48 hrs after reactivation with 10 ng/ml TNFα of SupT1 cells previously transduced with 1.00 x10^5^ pg of HIV-2 eGFP. Cells were pre-treated with increasing concentrations of GS-9822. Full lines represent non-activated cells, dotted lines represent TNFα treated cells. Data show one representative experiment out of 3 and the averages of duplicate measurements with standard deviation are plotted. a) Percentage of eGFP positive cells, b) Fold increase in eGFP positive cells (%eGFP^+^ TNFα treated cells/ %eGFP^+^ untreated cells), c-d) Summary data of 5 experiments of 1x10^5^ SupT1 cells transduced with 5.55 x10^3^ - 1.52 x10^4^ pg HIV-2 eGFP and pre-treated with GS-9822. Each data point represents the mean and SD from one experiment, with one color per experiment. Transduction was done in the same way for all experiments, but for 2 experiments reactivation was performed on day 8 instead of day 10, c) Fold increase in eGFP positive cells, calculated as previously described, at 24h post-reactivation, d) Fold increase in eGFP positive cells, calculated as previously described, at 48h post-reactivation.

#### Supplementary figure 5
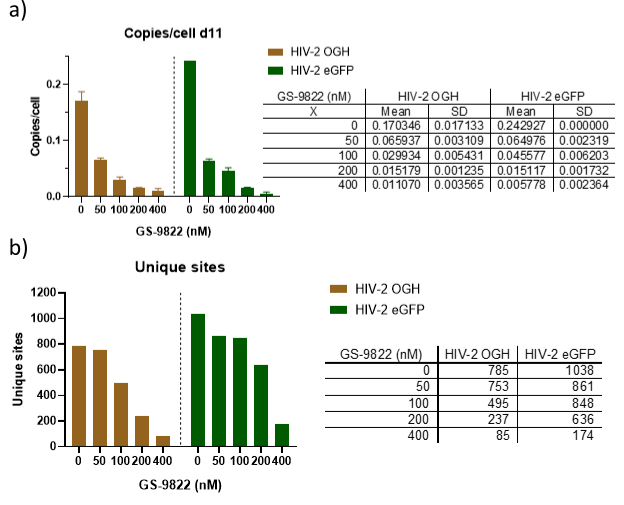


Figure V GS-9822 inhibits integration of HIV-2 OGH or HIV-2 eGFP transduced cells.
a) Number of integrated copies/cells determined on genomic DNA of NGS samples using HIV-2 Gag PCR and CCR5 qPCR, b) Number of unique sites as determined by NGS analysis

#### Supplementary figure 6


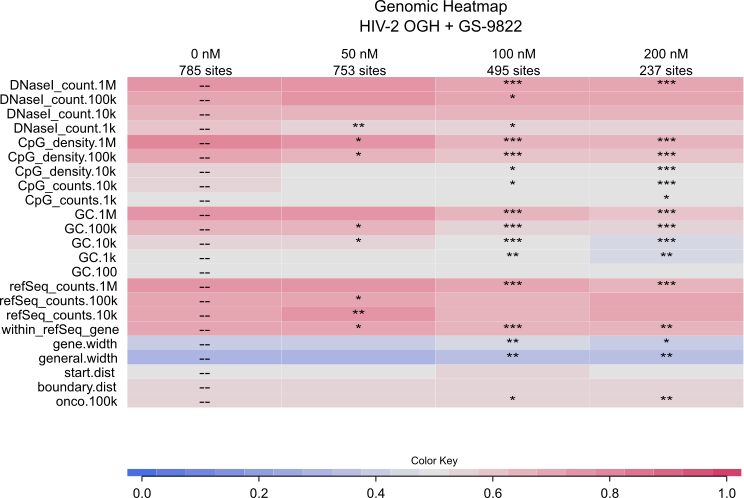


Figure VI GS-9822 retargets HIV-2 integration away from gene dense regions.
We transduced 400 000 SupT1 cells with 8.82 x10^3^ pg of HIV-2 OGH in the presence or absence of increasing concentrations of GS-9822 and kept in culture for at least 10 days. Next, genomic DNA was extracted for Illumina Miseq integration site sequencing and data were analyzed with the INSPIIRED software and represented as a heat map(58, 59). Colors indicate whether a genomic feature is favored (red) or disfavored (blue) for integration as compared to computer generated matched random controls (MRCs) using a receiver operating characteristic (ROC) curve area. Each integration site is compared to its MRC and according to the rank of the integration site, a number is assigned (1 if the feature is favored at the integration site over the MRC, 0 if it is disfavored and 0.5 if the feature is equal for the two sites). The analysis was done for all integration sites after which an average was calculated. These ROC curve areas are then statistically analyzed using Wald type test statistics which are referred to the Chi Square distribution (*=p<0.05, **p=0.01, ***=p<0.0001).

#### Supplementary figure 7


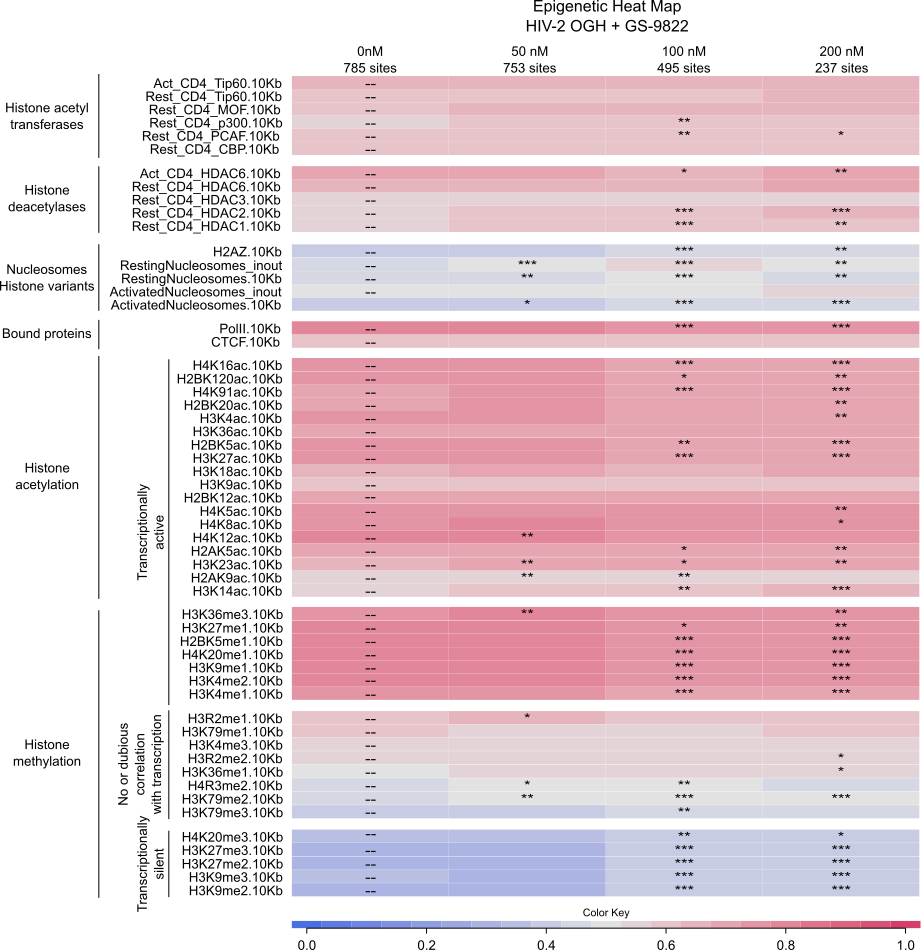


Figure VII Heat map representation of epigenetic features of HIV-2 OGH integration after treatment with GS-9822.
After Illumina Miseq sequencing, data were analyzed using the INSPIIRED software, yielding the heat map shown (58, 59). Color codes are as described in Fig. 5. The ROC curve areas were statistically analyzed using Wald type test statistics which are referred to the Chi Square distribution (*=p<0.05, **p=0.01, ***=p<0.0001).

#### Supplementary figure 8

Figure VIII Alignment of integrase amino acid sequences.
NCBI protein blast was used to align the integrase amino acid sequences of HIV-1 (subtype B isolate BH10), HIV-2 (subtype A, strain ROD) and SIVmac (strain k6W) protein sequences. Sequences were obtained from the Uniprot database and Uniprot entry numbers are listed for each integrase. Important residues for the LEDGF/p75 integrase interaction are highlighted in cyan if conserved between the integrases and in magenta when different.
